# Partial Cardiac Denervation to Prevent Postoperative Atrial Fibrillation After Coronary Artery Bypass Grafting: The pCAD-POAF Randomized Clinical Trial

**DOI:** 10.1001/jamacardio.2024.4639

**Published:** 2024-11-17

**Authors:** Ziang Yang, Xieraili Tiemuerniyazi, Fei Xu, Yang Wang, Yang Sun, Peng Yan, Liangxin Tian, Chao Han, Yan Zhang, Shiwei Pan, Zhan Hu, Xi Li, Wei Zhao, Wei Feng

**Affiliations:** 1 Department of Cardiovascular Surgery, Fuwai Hospital, National Center for Cardiovascular Diseases, National Clinical Research Center for Cardiovascular Diseases, Chinese Academy of Medical Sciences and Peking Union Medical College, Beijing, China; 2 Department of Medical Research and Biometrics Center, State Key Laboratory of Cardiovascular Disease, Fuwai Hospital, National Center for Cardiovascular Diseases, Chinese Academy of Medical Sciences and Peking Union Medical College, Beijing, China; 3 Department of Pathology Diagnostic Laboratory Service, Fuwai Hospital, National Center for Cardiovascular Diseases, Chinese Academy of Medical Sciences and Peking Union Medical College, Beijing, China; 4 State Key Laboratory of Cardiovascular Disease, Fuwai Hospital, National Center for Cardiovascular Diseases, National Clinical Research Center for Cardiovascular Diseases, Chinese Academy of Medical Sciences and Peking Union Medical College, Beijing, China

## Abstract

**Importance:**

Efficient approaches to prevent postoperative atrial fibrillation (POAF) after coronary artery bypass grafting (CABG) are still needed.

**Objective:**

To investigate whether partial cardiac denervation, achieved by cutting off the ligament of Marshall (LOM) and resecting the fat pad along the Waterston groove, can reduce the risk of POAF following CABG.

**Design, Setting and Participants:**

This single-center, randomized clinical trial enrolled adult patients scheduled for isolated CABG in China. Enrollment was from August 15, 2022, to December 13, 2023; follow-up visits were 30 days after discharge.

**Interventions:**

Participants were randomized into the intervention group (CABG plus partial cardiac denervation) and the control group (CABG only) in a 1:1 pattern. All participants were continuously monitored for the incidence of POAF until day 6 after the operation.

**Main outcome and Measures:**

The primary end point was the incidence of POAF in 6 days, defined as a supraventricular arrhythmia lasting for more than 30 seconds.

**Results:**

The trial enrolled 430 patients (79 [18.4%] female; mean [SD] age, 61.9 [7.8] years). Compared with the control group, the 6-day incidence of POAF was significantly lower in the intervention group (18.1% vs 31.6%; *P* = .001; risk ratio, 0.57 [95% CI, 0.41-0.81]). To further support these results, a sensitivity analysis performed with Kaplan-Meier survival curves also showed a significant reduction in the occurrence of POAF in the intervention group (hazard ratio, 0.53 [95% CI, 0.36-0.79]; *P* = .002). Safety assessments showed no difference between the 2 groups, while postoperative medical cost was reduced in the intervention group.

**Conclusions and Relevance:**

This randomized clinical trial found that partial cardiac denervation was an effective procedure to reduce the occurrence of POAF after isolated CABG without additional postoperative complications. These results suggest that partial cardiac denervation may be a good option for cardiac surgeons to consider for preventing POAF after CABG.

**Trial Registration:**

ClinicalTrials.gov Identifier: NCT05009914

## Key Points

**Question:**

Among patients undergoing isolated coronary artery bypass grafting (CABG), does a partial cardiac denervation procedure reduce the incidence of postoperative atrial fibrillation (POAF)?

**Findings:**

In this randomized clinical trial of 430 patients undergoing isolated CABG, partial cardiac denervation, achieved by cutting off the ligament of Marshall and resecting the fat pad along the Waterston groove, reduced the incidence of POAF without an increase in complications

**Meaning:**

This study’s results suggest that partial cardiac denervation may be an effective approach for cardiac surgeons to prevent POAF after CABG .

## Teaser

This randomized clinical trial examines whether a partial cardiac denervation procedure reduces the incidence of postoperative atrial fibrillation among patients undergoing isolated coronary artery bypass grafting (CABG) in China.

## Introduction

Postoperative atrial fibrillation (POAF) is a common complication after coronary artery bypass grafting (CABG), which occurs most commonly within 1 week after surgery, with an incidence ranging from 5% to 40%.^1^ POAF may prolong the length of hospitalization and elevate the risk of stroke and mortality, thereby increasing medical costs.^3^ Furthermore, POAF might also increase the long-term risk of cumulative cerebrovascular accidents.^5^

The cardiac autonomic nerve system (CANS) plays a vital role in the development of POAF.^6^ Nevertheless, the prevalence of POAF following CABG still stands at 21.1% despite the widespread use of β-blockers, which inhibit cardiac sympathetic excitability and are the sole medication recommended as a class I treatment by the current guidelines to prevent POAF.^7^ The intrinsic CANS mainly consists of ganglia and relative nerves found in the epicardial adipose tissue (EAT) surrounding the cardiac major arteries and veins, including the ligament of Marshall (LOM) and the fat pad along the Waterston groove.^9^ The activation of CANS embedded in the EAT is considered to take part in the development of atrial fibrillation (AF), which is the main theory for cutting off the LOM and resecting the fat pad along the Waterston groove during Maze procedure for the treatment of AF.^12^ Therefore, we hypothesized that partial cardiac denervation through these 2 sites might be useful for reducing the risk of POAF after CABG. By cutting off the LOM and resecting the fat pad along the Waterston groove, this trial aimed to test the hypothesis that partial cardiac denervation could reduce POAF after isolated CABG.

## Methods

### Study Design and Patient Eligibility Criteria

This single-center, prospective, randomized clinical trial was conducted according to the Declaration of Helsinki, approved by the institutional review board of Fuwai Hospital, and registered at ClinicalTrials.gov in June 2021 (protocol record: NCRC2020003; and identifier: NCT05009914). Reporting of this trial followed the Consolidated Standards of Reporting Trials (CONSORT) guideline. The study was overseen by both the institutional review board and the data monitoring committee for safety and efficacy concerns during the entire process. The study protocol was previously published.^13^

Adult patients scheduled for isolated CABG were screened for eligibility. Exclusion criteria were as follows: (1) age younger than 18 years; (2) urgent CABG; (3) prior cardiac surgery history; (4) concurrent cardiac surgery (such as repair of congenital heart disease, left ventricular reconstruction, valvular surgery, or surgery for aortic diseases); (5) critical condition requiring mechanical or pharmaceutical sustainment before CABG; (6) AF history (defined as supraventricular heart rhythm disorder characterized by rapid and irregular electrical activity in the atrium); and (7) taking antiarrhythmic medications other than β-blockers in 2 weeks before the surgery.

### Randomization

After giving written informed consent, a computer-generated minimized random allocation approach was used to randomly assign individuals. As POAF is strongly associated with various factors,^6^ including age, left ventricular systolic function, a history of AF and left atrial enlargement, to ensure a proper baseline balance after randomization, randomization was stratified by age, left ventricular ejection fraction (LVEF), and history of myocardial infarction (eMethods 1 in Supplement 1). All surgeons were informed of the patient’s specific group assignment once their patients were under general anesthesia in the operating room.

### Interventions

Before scheduled CABG, the intervention group underwent a partial cardiac denervation procedure achieved by cutting off the LOM and resecting the fat pad along the Waterston groove. If the patient’s hemodynamics was unstable by the judgement of the surgeon during off-pump CABG, the partial cardiac denervation procedure was performed after anastomosis of grafts. The specific procedure was described previously^13^ and detailed in eMethods 2 in Supplement 1. The anatomic sites of the LOM and the fat pad along the Waterston groove were shown in eFigures 1 and 2 in Supplement 1. Thorough cardiac denervation to the surface of myocardium was necessary. In the control group, patients underwent isolated CABG without an additional surgical procedure.

At the beginning, all surgeons taking part in this trial observed how the intervention procedure was performed by 2 experienced chief surgeons. After completing no more than 3 procedures with their guidance, all of the surgeons could solely and proficiently master this technique.

To confirm the existence of intrinsic CANS in the resected EAT and that partial cardiac denervation was achieved, 10 pairs of Waterston fat pad samples from participants (1 with and 1 without POAF in each pair) were selected to perform histologic analysis. All samples were fixed in formalin and cut (from myocardial to epicardial side) into 2 or 3 segments according to their size. Each segment was embedded in paraffin, sliced into sections and stained by hematoxylin-eosin. The example of the fat pad along the Waterston groove and the example of histologic results are presented separately in eFigures 3 and 4 in Supplement 1. Ganglia and/or nerve fibers could be seen in all samples (eTables 1 and 2 in Supplement 1).

### Outcomes

By wearing the NS-SP-B-01 Attached Dynamic Electrocardiogram (ECG) Recording System (Ensense, Shanghai, China), all participants were continuously monitored from within 1 hour after the surgery to postoperative day 6. Additional 12-lead ECGs would be conducted if necessary. After the surgery, all of the patients were prescribed β-blockers unless there were complications with contradictions such as bradycardia or atrioventricular block. Participants were assessed by 12-lead standard ECG and echocardiogram on the day of discharge. The 30-day follow-up after discharge was completed by outpatient visit and/or phone calls.

The primary outcome was the occurrence of POAF in 6 days, defined as a supraventricular arrhythmia lasting for more than 30 seconds.^14^ Additionally, the number of AF episodes and AF burden (defined as the ratio of total AF time to recording time) were compared between the 2 groups.

The secondary outcomes included both perioperative and follow-up events. These were: (1) safety assessments including the incidence of transferring to on-pump CABG, secondary operation for bleeding, requirement for blood transfusions, delayed pericardial effusion, and critical arrhythmias except AF within 30 days after discharge; (2) economic assessments of the postoperative length of hospitalization and postoperative medical cost; and (3) major adverse cardiovascular and cerebrovascular events (MACCE), which were defined as the composite of stroke, myocardial infarction, repeat coronary revascularization and all-cause mortality during the 30-day follow-up. Delayed pericardial effusion was defined as new-onset pericardial effusion (moderate or more) within 30 days after discharge. Critical arrhythmias other than AF were defined as arrhythmias requiring immediate clinical intervention, such as complete atrioventricular block.

### Statistical Analysis

We assumed a rate of POAF of 23% after cardiac surgery according to previous studies and that cardiac denervation would reduce the occurrence by 50%.^16^ An estimation of 408 participants were required to provide 80% power and .05 α (2-sided). Considering a certain rate of protocol violation, participant withdrawal and loss to follow-up, a total of 430 participants (215 in each group) was sufficient for this trial.

All analyses were conducted according to the intention-to-treat principle. Continuous variables were expressed as mean (SD) or median (IQR), and tested by *t* test or Mann-Whitney *U* test according to their distributions. Categorical variables were presented as numbers (%) and tested by χ^2^ test or by Fisher exact test, as appropriate. To further confirm the results, a sensitivity analysis regarding the primary outcome was carried out by the time-to-event analysis performed with Kaplan-Meier survival curves and compared by the log-rank test.

Subgroup analysis was conducted based on the risk factors of POAF reported by previous studies, including on/off-pump, sex, age (≥65 years vs <65 years), LVEF (>55% vs ≤55%), body mass index (calculated as weight in kilograms divided by height in meters squared; ≥25 vs <25), left atrium size (≥40 mm vs <40 mm), and history of myocardial infarction, diabetes, and hypertension. Two-tailed *P* < .05 was considered statistically significant. Statistical analyses were performed using R 4.0.2 (R Core Team) and Stata 15.0 (StataCorp).

## Results

**Figure 1.**
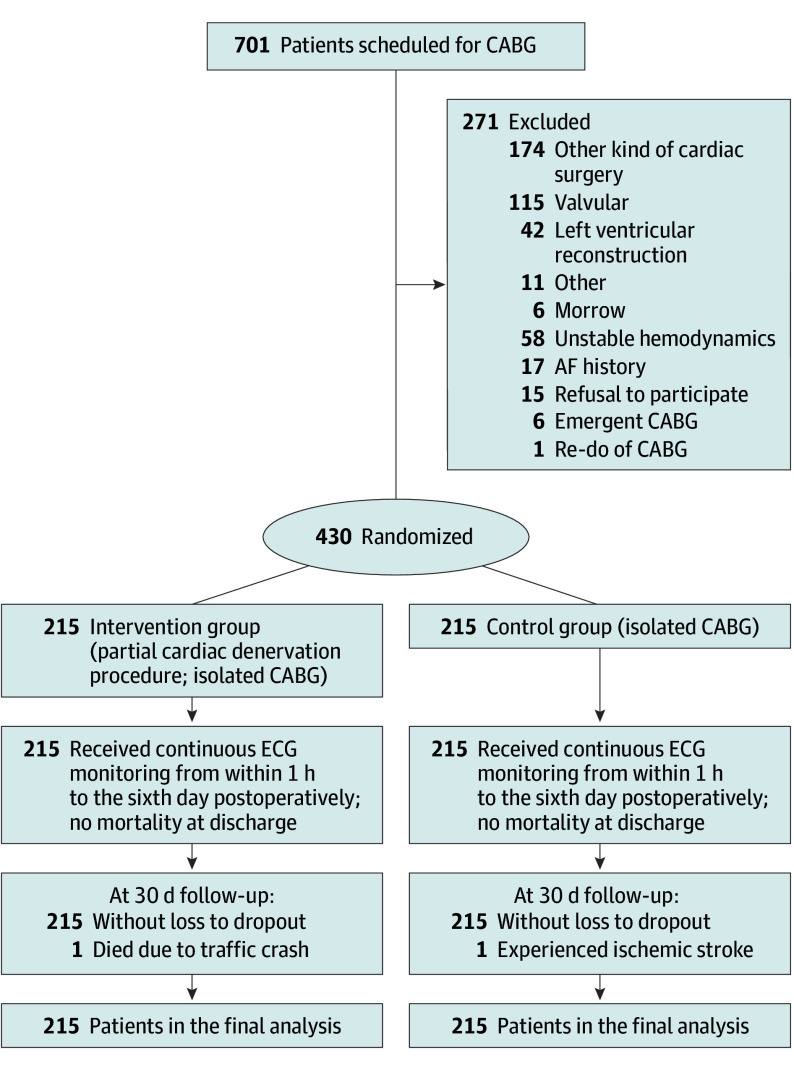
Flowchart of the Study AF indicates atrial fibrillation; CABG, coronary artery bypass grafting; ECG, electrocardiogram.

From August 15, 2022, to December 13, 2023, a total of 701 patients were screened for eligibility, and 430 patients were enrolled in this study (Figure 1). The mean (SD) age was 61.9 (7.8) years, and 79 participants (18.4%) were female. Baseline characteristics were well balanced after randomization between the intervention group (n = 215) and the control group (n = 215) (Table 1).

**Table 1.** Baseline and Intraoperative Characteristics

Variables	Control (n = 215)	Intervention (n = 215)
Baseline		
Age, mean (SD), y	61.9 (7.6)	61.9 (7.9)
Sex, No. (%)		
Female	40 (18.6)	39 (18.1)
Male	175 (81.4)	176 (81.9)
BMI, median (IQR)	26.0 (23.8-28.0)	25.9 (24.1-27.8)
NYHA III or IV, No. (%)	35 (16.3)	37 (17.2)
Hypertension, No. (%)	165 (76.7)	152 (70.7)
Diabetes, No. (%)	93 (43.3)	108 (50.2)
Insulin users, No. (%)	31 (14.4)	40 (18.6)
Dyslipidemia, No. (%)	178 (82.8)	173 (80.5)
Chronic kidney dysfunction, No. (%)	2 (0.9)	3 (1.4)
Left main disease, No. (%)	42 (19.5)	36 (16.7)
Triple vessel diseases, No. (%)	188 (87.4)	183 (85.1)
Myocardial infarction, No. (%)	85 (39.5)	85 (39.5)
Prior PCI, No. (%)	41 (19.0)	49 (22.8)
Stroke, No. (%)	39 (18.1)	42 (19.5)
Peripheral arterial disease, No. (%)	40 (18.6)	32 (14.9)
Chronic lung disease, No. (%)	1 (0.5)	3 (1.4)
Smoking, No. (%)	107 (49.8)	110 (51.2)
NT-pro BNP, median (IQR), pg/mL	121.0 (59.9-302.0)	115.0 (51.9-239.0)
T3, median (IQR), ng/mL	1.1 (1.0-1.2)	1.1 (1.0-1.2)
hsCRP, median (IQR), mg/L	1.0 (0.5-2.7)	1.0 (0.4-3.2)
ALT, median (IQR), IU/L	21.0 (16.0-29.0)	22.0 (14.0-35.0)
AST, median (IQR), IU/L	22.0 (17.0-28.0)	23.0 (17.0-30.0)
Creatinine, median (IQR), μmol/L	68.4 (59.5-77.9)	67.3 (58.8-78.1)
LVEF, median (IQR)	63.0 (60.0-66.0)	63.0 (60.0-67.0)
Left atrium, median (IQR), mm	36.0 (34.0-40.0)	36.0 (34.0-38.0)
LVEDD, median (IQR), mm	48.0 (45.0-52.0)	48.0 (45.0-51.0)
Mitral regurgitation, No. (%)		
No/trivial	159 (74.0)	173 (80.5)
Mild	56 (26.1)	42 (19.5)
β-Blocker, No. (%)	188 (87.4)	188 (87.4)
Intraoperative		
On-pump, No. (%)	91 (42.3)	86 (40)
CPB duration, median (IQR), min	107.0 (82.0-133.0)	102.0 (80.0-123.0)
Cross clamp time, median (IQR), min	73.0 (57.0-95.0)	72.5 (55.0-89.0)
Operation time, median (IQR), min	234.0 (200.0-260.0)	228.0 (203.0-255.0)
No. of grafts, mean (SD)	3.8 (0.8)	3.7 (0.8)
Arterial grafts, mean (SD)	1.1 (0.4)	1.0 (0.3)
Venous grafts, mean (SD)	2.7 (0.8)	2.7 (0.9)

Abbreviations: ALT, alanine transaminase; AST, aspartate transaminase; BMI, body mass index (calculated as weight in kilograms divided by height in meters squared); CPB, cardiopulmonary bypass; hs-CRP, high-sensitivity C-reactive protein; LVEDD, left ventricular end diastolic diameter; LVEF, left ventricular ejection fraction; NT-pro BNP, N-terminal B-type natriuretic peptide; NYHA, New York Heart Association; PCI, percutaneous coronary intervention.

SI conversion factors: To convert ALT to μkat/L, multiply 0.0167; AST to μkat/L, multiply 0.0167; creatinine to mg/dL, divide by 88.4; hs-CRP to mg/dL, divide by 10; NT-pro BNP to ng/L, multiply by 1.

All participants underwent scheduled CABG successfully. The partial cardiac denervation procedure did not significantly increase total operative time or bypass time (Table 1) and was successfully performed on each patient in the intervention group. Nineteen patients who underwent off-pump CABG emerged with a transient increase in heart rate when resecting the fat pad along the Waterston groove. No other complications or adverse events occurred during the partial cardiac denervation procedure.

There were no in-hospital deaths in either group. One patient in the intervention group and 3 in the control group developed new-onset stroke, respectively. For blood inflammatory biomarkers, the level of postoperative interleukin (IL)-4, IL-6, IL-8, tumor necrosis factor α and high-sensitivity C-reactive protein of the patients were comparable between the intervention and control group (eTable 3 in Supplement 1).

### Primary Outcome

**Figure 2.**
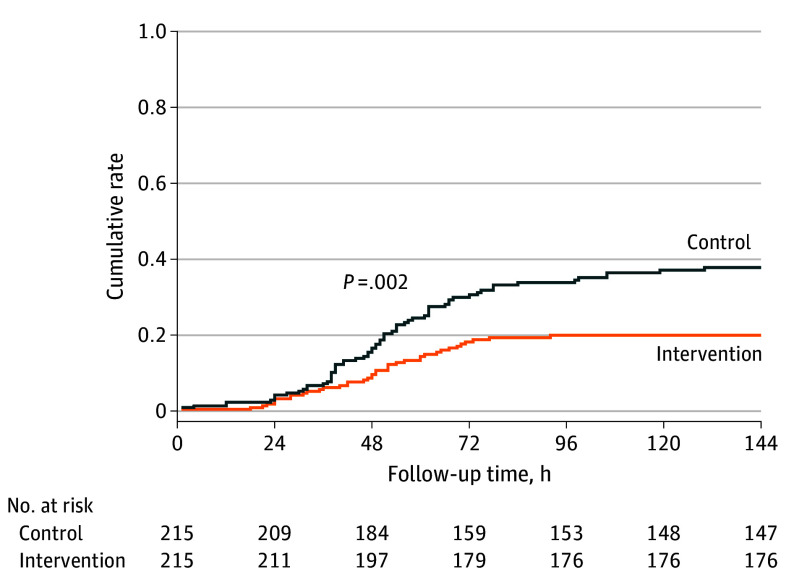
Kaplan-Meier Curves of the Primary Outcome

All patients were included in the final analysis. The postoperative use of β-blockers did not differ in each group (206 [95.8%] vs 200 [93.0%]; *P* = .21). Thirty-nine patients in the intervention group and 68 patients in the control group developed POAF, respectively. As compared with the control group, partial cardiac denervation reduced the risk of POAF (18.1% vs 31.6%; *P* = .001; risk ratio: 0.57 [95% CI, 0.41-0.81]). In the sensitivity analysis, the Kaplan-Meier analysis also showed a significant reduction in the occurrence of POAF in the intervention group (hazard ratio, 0.53 [95% CI, 0.36-0.79]; *P* = .002) (Figure 2).

Subgroup analysis for the primary outcome was conducted in terms of potential risk factors. The results showed that partial cardiac denervation had similar treatment effect in each subgroup (eFigure 5 in Supplement 1). The incidence of POAF was similar between on-pump (30.8%) and off-pump CABG (31.5%) in the control group.

### POAF-Related Measurements

Median (IQR) AF episodes were significantly reduced in the intervention group (0 [0-0] vs 0 [0-3]; *P* = .002), with a maximum of 223 in the intervention group and 158 in the control group, respectively. AF burden was lower in the intervention group (AF burden of 0%: 147 [68.4%] vs 176 [81.9]; >0% to ≤10%: 58 [27.0%] vs 31 [14.4%]; >10%: 10 [4.7%] vs 8 [3.7%]; *P* = .004) (Table2), while 2 patients in the control group were diagnosed with POAF that lasted more than half of the monitoring time, detailed as 99.97% and 100%. These were the only 2 patients with persistent AF on the day of discharge.

**Table 2.** POAF-Related Measurements

Variables	Control (n = 215)	Intervention (n = 215)	*P* value
AF burden, No. (%)			
0	147 (68.4)	176 (81.9)	.004
>0 and ≤10%	58 (27.0)	31 (14.4)	
>10%	10 (4.7)	8 (3.7)	
POAF lasting time, No. (%)			
0	146 (67.9)	175 (81.4)	.01
>0 and ≤6 min	5 (2.3)	2 (0.9)	
>6 min and ≤6 h	38 (17.7)	22 (10.2)	
>6 h and ≤24 h	23 (10.7)	11 (5.1)	
>24 h	3 (1.4)	5 (2.4)	
AF episodes, No. (%)	0 (0 to 3)	0 (0 to 0)	.002
Antiarrhythmic therapy			
β-Blocker, No. (%)	200 (93.0)	206 (95.8)	.21
Cardioversion, No. (%)	0	0	NA
Amiodarone, No. (%)	57 (26.5)	31 (14.4)	.002
% of Premature atrial contractions, median (IQR)	0.044 (0.007 to 0.489)	0.026 (0.007 to 0.233)	.47
% of Single premature atrial contractions, median (IQR)	0.041(0.005 to 0.397)	0.024 (0.007 to 0.200)	.42
% of Couplets, median (IQR)	0.001 (0 to 0.019)	0.001 (0 to 0.005)	.42
% of Nonsustained atrial tachyarrhythmias, median (IQR)	0.002 (0 to 0.023)	0.001 (0 to 0.008)	.31
Highest HR, median (IQR), beats/min	146 (125 to 182)	133 (119 to 166)	.002
Lowest HR, median (IQR), beats/min	56 (49 to 61)	59 (52 to 65)	.001
Mean HR, median (IQR), beats/min	81 (77 to 87)	83 (78 to 88)	.24
Acceleration capacity, median (IQR), ms	−3.1 (−2.7 to −3.8)	−2.8 (−2.4 to −3.4)	<.001
Deceleration capacity, median (IQR), ms	2.8 (2.3 to 3.4)	2.6 (2.2 to 3.1)	.008

Abbreviations: AF, atrial fibrillation; HR, heart rate; NA, not applicable; POAF, postoperative atrial fibrillation.

There was no difference regarding the percentage of single premature atrial contractions, couplets, nonsustained atrial tachyarrhythmias and mean heart rate between the 2 groups postoperatively. However, the acceleration capacity and the deceleration capacity were different between the 2 groups, presenting a potential autonomic modulation (Table 2).

### Secondary Outcomes

For the safety assessments, the incidence of perioperative blood transfusion (33 [15.4%] vs 44 [20.5%]; *P* = .17), reoperation for postoperative bleeding (0 [0%] vs 2 [0.9%]; *P* = .50), and delayed pericardial effusion (1 [0.5%] vs 2[0.9%]; *P* > .99) was similar between groups (Table 3). No patient transferred to on-pump CABG intraoperatively or developing critical arrhythmias within the 30-day follow-up. However, 1 patient in each group developed AF after discharge.

**Table 3.** Secondary Outcomes

Variables	Control (n = 215)	Intervention (n = 215)	*P* value
Perioperative			
Blood transfusion, No. (%)	44 (20.5)	33 (15.4)	.17
Reoperation for bleeding, No. (%)	2 (0.9)	0	.50
Postoperative length of hospitalization, mean (SD), days	7.3 (3.3)	6.8 (1.8)	.09
Medical cost, median (IQR), US $	4052.9 (3338.2-5278.8)	3909.2 (3226.5-4917.9)	.05
Follow-up			
Delayed epicardial effusion, No. (%)	2 (0.9)	1 (0.5)	>.99
MACCE within 30 d, No. (%)	1 (0.5)	1 (0.5)	>.99

Abbreviation: MACCE, major adverse cardiovascular and cerebrovascular events.

For the economic assessments, the postoperative length of hospitalization was a mean (SD) of 6.8 (1.8) days in the intervention group vs 7.3 (3.3) days in the control group (*P* = .09) (Table 3). Patients in the intervention group had shorter median (IQR) intensive care unit stay times than the control group (21.0 [17.0-42.0] vs 22.0 [17.0-51.0] hours; *P* = .05) (eTable 3 in Supplement 1). The median (IQR) cost was also reduced in the intervention group (US $3909.2 [US $3226.5-4917.9] vs US $4052.9 [US $3338.2-5278.8]; *P* = .05).

Within the 30-day follow-up, 1 patient in the intervention group died of traffic accident and 1 patient in the control group suffered from ischemic stroke. There was no difference of MACCE between each group (1 [0.5%] vs 1 [0.5%]; *P* > .99) (Table 3).

## Discussion

In this randomized clinical study, partial cardiac denervation reduced the occurrence of POAF by 47%, as well as the AF burden and number of AF episodes after isolated CABG. This procedure was cost-efficient, because patients in the intervention group had shorter intensive care unit stays and less postoperative medical costs without additional complications.

The mechanisms of POAF after cardiac surgery are extremely complicated and remain unclear. Generally, the development of POAF is based on 3 aspects,^6^ including (1) the atrial remodeling substrate, such as left atrial enlargement and fibrosis, which is associated with aging, hypertension, and genetics; (2) surgery-induced substrate resulting from cardiopulmonary bypass or atriotomy; and (3) transient postoperative factors such as CANS, inflammation, or oxidative stress.

Numerous research studies have investigated the effects of autonomic neuromodulation therapies and surgical means to avert POAF. Several studies attempted to prevent POAF by excising the fat pads surrounding the heart’s major vessels and injecting botulinum toxin into the epicardial fat pads.^19^ The results were inconsistent due to limited sample size and nonoptimal trial design. Wang et al^15^ tried to suppress the function of 4 major ganglionated plexi by injecting calcium chloride and reported a reduction of POAF. However, this study only enrolled patients undergoing off-pump CABG and the injection procedure required a certain learning curve. The NOVA study^24^ also showed similar outcomes, though the sample size was limited. As the largest study on this clinical field, the current pCAD-POAF trial enrolled either on-pump or off-pump patients. In this study, partial cardiac denervation was achieved by surgical resection of LOM and fat pad along the Waterston groove. The results showed that the incidence of POAF, AF episodes, and AF burden were reduced after the partial cardiac denervation procedure, which meanwhile did not significantly increase the operation time, cause bleeding or critical arrhythmias. Although the postoperative length of hospital stay was not statistically different, partial cardiac denervation still brought economic benefit and decreased the intensive care unit stay.

One major concern may be the safety of the procedure. In our study, there was no difference between the 2 groups regarding the percentage of premature atrial contractions (single/couplets/nonsustained atrial tachyarrhythmias), mean heart beats, echocardiogram parameters at discharge and the 30 days follow-up visit (eTable 3 in Supplement 1). Even though no postoperative complications were observed, long-term follow-up to assess patients’ performance such as exercise tolerance and functional capacity, is still needed to fully evaluate the safety and long-term outcomes of this procedure.

It is believed that the stimulation of sympathetic tone may lead to POAF, because higher levels of norepinephrine and more administration of inotropic agents were found in patients with POAF.^25^ Nevertheless, both higher and lower heart rate variability could be seen before POAF,^27^ suggesting that dysfunction of not only sympathetic but also vagal tone may participate in the process.^28^ With the histologic results in this trial, we confirm the existence of ganglia and nerve fibers in the EAT we resected and the preventive role of partial cardiac denervation. However, the exact interactions between CANS and myocardium, as well as how the balance of sympathetic and vagal tone changes remain uncertain.

There is scarce evidence directly derived from human samples to provide information of the postoperative change in patients with POAF. Most studies tried to associate blood inflammatory biomarkers such as IL-4 and IL-6 with POAF, but the results were inconsistent.^29^ In our study, these results were comparable between the 2 groups. Under this circumstance, the local ganglia regulation and inflammatory effect of EAT may play a vital role in the development of POAF.

Cutting off the LOM and resecting the fat pad along the Waterston groove was not a highly-demanding technique and could be mastered easily after a short-term training. Therefore, surgical partial cardiac denervation might be an effective and convenient approach for cardiac surgeons to prevent POAF after CABG.

### Limitations

Our study has limitations. First, due to the impact of COVID-19 pandemic, we encountered challenges in conducting a multicenter trial, thus heterogeneity across cardiac surgery centers in implementing the intervention has not been examined. Second, given that POAF predominantly manifests within the first week after surgery, our study primarily focused on outcomes during hospitalization and short-term follow-up. However, it remains imperative to conduct long-term follow-ups to thoroughly investigate the safety and efficacy of this partial cardiac denervation technique. We intend to invite all patients to participate in a long-term assessment after 1 year or more in the future.

## Conclusions

This RCT found that the occurrence of POAF after isolated CABG could be efficiently reduced by partial cardiac denervation through cutting off the LOM and resecting the fat pad along the Waterston groove. These results suggest that partial cardiac denervation may be viable option for cardiac surgeons to consider for reducing the risk of POAF after CABG

## Notes

Supplement 1. **eMethods 1.** Details of the Randomization Scheme **eMethods 2.** Partial Cardiac Denervation Procedure **eTable 1.** Histologic Results of the Waterston fat Pad Resected From Patients Who Developed POAF **eTable 2.** Histologic Results of the Waterston Fat Pad Resected From Patients Without POAF **eTable 3.** Other Postoperative and Follow-Up Results **eFigure 1.** Anatomic Site of the LOM **eFigure 2.** Anatomic Site of the Fat Pad Along the Waterston Groove **eFigure 3.** Example of the Fat Pad Along the Waterston Groove **eFigure 4.** Examples of the Histologic Results Performed on the Fat Pad Along the Waterston Groove **eFigure 5.** Subgroup Analysis for Primary Outcome

Supplement 2.
Trial Protocol and Statistical Analysis Plan

Supplement 3.
Data Sharing Statement
