# Supplementary material for: Partial Cardiac Denervation to Prevent Postoperative Atrial Fibrillation After Coronary Artery Bypass Grafting: The pCAD-POAF Randomized Clinical Trial
Source: JAMA Cardiol. 2024 Nov 17:e244639. Online ahead of print. doi: 10.1001/jamacardio.2024.4639 (PMC11571071; doi:10.1001/jamacardio.2024.4639)
Supplement: Supplement 1. — eMethods 1. Details of the Randomization Scheme eMethods 2. Partial Cardiac Denervation Procedure eTable 1. Histologic Results of the Waterston fat Pad Resected From Patients Who Developed POAF eTable 2. Histologic Results of the Waterston Fat Pad Resected From Patients Without POAF eTable 3. Other Postoperative and Follow-Up Results eFigure 1. Anatomic Site of the LOM eFigure 2. Anatomic Site of the Fat Pad Along the Waterston Groove eFigure 3. Example of the Fat Pad Along the Waterston Groove eFigure 4. Examples of the Histologic Results Performed on the Fat Pad Along the Waterston Groove eFigure 5. Subgroup Analysis for Primary Outcome [file jamacardiol-e244639-s001.pdf]

Supplemental Online Content

Yang Z, Tiemuerniyazi X, Xu F, et al. Partial cardiac denervation to prevent postoperative atrial fibrillation after coronary artery bypass grafting: the pCAD-POAF randomized clinical trial. *JAMA Cardiol.* Published online November 17, 2024. doi:10.1001/jamacardio.2024.4639

eMethods 1. Details of the Randomization Scheme

eMethods 2. Partial Cardiac Denervation Procedure

eTable 1. Histologic Results of the Waterston fat Pad Resected From Patients Who Developed POAF

eTable 2. Histologic Results of the Waterston Fat Pad Resected From Patients Without POAF

eTable 3. Other Postoperative and Follow-Up Results

eFigure 1. Anatomic Site of the LOM

eFigure 2. Anatomic Site of the Fat Pad Along the Waterston Groove

eFigure 3. Example of the Fat Pad Along the Waterston Groove

eFigure 4. Examples of the Histologic Results Performed on the Fat Pad Along the Waterston Groove

eFigure 5. Subgroup Analysis for Primary Outcome

This supplemental material has been provided by the authors to give readers additional information about their work.

© 2024 Yang Z et al. *JAMA Cardiology.*

eMethods 1. Details of the randomization scheme.

The randomization scheme was stratified by age (< 50, ≥ 50 and < 60, ≥ 60 and <70, or ≥ 70), sex (female or male), left ventricular ejection fraction (LVEF) (< 40, ≥ 40 and < 50, ≥ 50 and <60, or ≥ 60), and history of myocardial infarction (yes or no).

eMethods 2. Partial cardiac denervation procedure.

In the intervention group, we performed partial cardiac denervation on both left and right atrium. The procedure was achieved by cutting off the LOM and resecting the fat pad along the Waterston groove. Specifically, during the on-pump CABG, the heart was pulled to the right to expose the LOM between the left atrial appendage and the left pulmonary veins under extracorporeal circulation, and the LOM was cut off by electrotome. Then, the fat pad along the Waterston groove was exposed between the right pulmonary veins and right atrium and excised completely to the surface of myocardium, with the upper edge extending beyond the opening of the right upper pulmonary vein and the lower edge to the inferior vena cava. For off-pump CABG, we cut off the LOM in the same way mentioned above. Then if the patient's heart was well tolerated and hemodynamics was stable, a fixator was used to fix the heart when resecting the fat pad along the Waterston groove. Otherwise, partial cardiac denervation was performed after the anastomosis of grafts.

© 2024 Yang Z et al. *JAMA Cardiology.*

eTable 1. Histologic results of the Waterston fat pad resected from patients who developed POAF. Number ①-③ stands for different segments from one sample. A-C stands for different 10× fields of view under microscope.

Samples from POAF patients								
Patient No.	Segment No.	Number of ganglia			Number of nerve fibers			
		A	B	C	A	B	C	
1	①	0	0	1	2	1	0	
	②	1	1	0	1	2	2	
	③	1	0	1	5	2	3	
2	①	0	0	0	0	1	2	
	②	0	0	0	2	2	1	
	③	2	1	1	1	2	1	
3	①	0	0	1	1	5	2	
	②	0	0	1	6	2	2	
	③	1	0	0	1	2	0	
4	①	0	0	0	2	3	2	
	②	1	0	0	1	0	2	
	①	0	0	0	2	5	4	

© 2024 Yang Z et al. *JAMA Cardiology.*

5	②	0	0	0		2	2	6
	③	0	0	0		6	5	0
6	①	0	0	0		5	3	2
	②	1	1	0		2	2	2
	③	0	0	0		3	2	4
7	①	0	0	0		3	4	1
	②	0	0	0		1	2	3
	③	0	0	0		1	4	3
8	①	0	1	1		2	2	2
	②	2	0	0		4	1	3
9	①	0	0	0		0	2	2
	②	0	0	0		3	3	0
	③	1	0	0		5	1	2
10	①	0	0	0		0	2	2
	②	0	0	0		3	3	0
	③	1	0	0		5	1	2

eTable 2. Histologic results of the Waterston fat pad resected from patients without

© 2024 Yang Z et al. *JAMA Cardiology.*

POAF. Number ①-③ stands for different segments from one sample. A-C stands for different 10× fields of view under microscope.

Samples from non-POAF patients								
Patient No.	Segment No.	Number of ganglia				Number of nerve fibers		
		A	B	C		A	B	C
11	①	0	0	0		1	0	1
	②	0	0	0		2	0	1
	③	0	0	0		3	1	1
12	①	1	0	0		5	2	4
	②	1	0	0		3	2	2
	③	1	1	0		5	3	2
13	①	0	0	0		1	2	3
	②	1	1	0		0	2	3
	③	1	0	0		2	2	3
14	①	0	0	0		1	1	1
	②	0	0	1		6	2	0
	③	0	0	0		2	1	0

© 2024 Yang Z et al. *JAMA Cardiology.*

15	①	0	0	0		1	1	2
	②	0	0	0		1	0	1
	③	1	0	0		0	1	1
16	①	1	0	1		0	2	1
	②	0	0	0		2	1	0
	③	1	0	0		1	0	4
17	①	1	0	1		1	4	3
	②	0	0	0		1	1	1
	③	2	0	0		1	1	2
18	①	0	0	0		2	1	2
	②	1	0	0		1	0	1
19	①	0	1	0		3	3	2
	②	0	0	2		2	4	1
	③	1	1	1		1	6	1
20	①	0	1	0		1	0	1
	②	1	0	0		1	3	2

© 2024 Yang Z et al. *JAMA Cardiology.*

	③	1	0	0		4	2	2

eTable 3. Other postoperative and follow-up results.

Variables	Control (n=215)	Intervention (n=215)	p -value
**Postoperative**			
NT-pro BNP, median [Q1, Q3], pg/mL	1552.0 [955.0, 2509.0]	1398.0 [932.0, 2300.0]	.15
hsCRP, median [Q1, Q3], mg/L	11.9 [10.8, 12.6]	11.8 [11.0, 12.5]	.82
IL-4, median [Q1, Q3], pg/mL	1.9 [1.2, 2.7]	1.9 [1.2 2.5]	.16
IL-6, median [Q1, Q3], pg/mL	167.2 [84.6, 352.0]	167.2 [101.4, 368.1]	.30
IL-8, median [Q1, Q3], pg/mL	31.9 [17.6, 59.1]	27.8 [15.0, 60.3]	.35
TNF-α, median [Q1, Q3], pg/mL	3.2 [1.7, 6.0]	3.2 [1.7, 5.0]	.39
IABP, no (%)	1 (0.5)	0	> .99
Intubation time, median [Q1, Q3], hours	8.0 [6.0, 13.0]	7.0 [6.0, 12.0]	.48
ICU stay, median [Q1, Q3], hours	22.0 [17.0, 51.0]	21.0 [17.0, 42.0]	.05

© 2024 Yang Z et al. *JAMA Cardiology.*

AKI, no (%)	55 (25.6)	45 (20.9)	.79
Stage 2 or 3	7 (3.3)	8 (3.7)	
New-onset stroke, no (%)	3 (1.4)	1 (0.5)	.62
Postoperative sternal infection, no (%)	6 (2.8)	6 (2.8)	> .99
Chest tube drainage, median [Q1, Q3], mL	1040.0 (840.0, 1365.0)	1040.0 (830.0, 1300.0)	.39
OAC at discharge, no (%)	11 (5.1)	4 (1.9)	.07
**Echocardiogram at discharge**			
LVEF, median [Q1, Q3]	60.0 [58.0, 64.0]	62.0 [58.0, 65.0]	.07
LVEDD, median [Q1, Q3], mm	45.0 [41.0, 49.0]	45.0 [42.0, 48.0]	.94
Mitral regurgitation, no (%)			.17
No/trivial	179 (83.3)	189 (87.9)	
Mild	36 (16.7)	26 (12.1)	
**Echocardiogram at follow-up***	**n=210**	**n=210**	
LVEF, median [Q1, Q3]	59.4 [55.0, 63.0]	59.0 [55.0, 63.0]	.95

© 2024 Yang Z et al. *JAMA Cardiology.*

LVEDD, median [Q1, Q3], mm	46.0 [43.0,50.0]	46.0 [43.0, 49.0]	.36
Mitral regurgitation, no (%)			.13
No/trivial	94 (44.8)	112 (53.3)	
Mild	108 (51.4)	95 (45.2)	
Moderate	7 (3.3)	3 (1.4)	
Severe	1 (0.5)	0	

AKI, acute kidney injury; hs-CRP, high-sensitivity C-reactive protein; IABP, intra-aortic balloon pump; ICU, intensive care unit; IL, interleukin; LVEDD, left ventricular end diastolic diameter; LVEF, left ventricular ejection fraction; NT-pro BNP, N-terminal B-type natriuretic peptide; OAC, oral anticoagulants; TNF-α, tumor necrosis factor-α; *5 patients in each group failed to provide follow-up echocardiogram results.

© 2024 Yang Z et al. *JAMA Cardiology.*

eFigure 1. Anatomic site of the LOM. LAA, left atrial appendage; LIMA, left internal mammary artery; LOM, ligament of Marshall; LPV, left pulmonary vein.

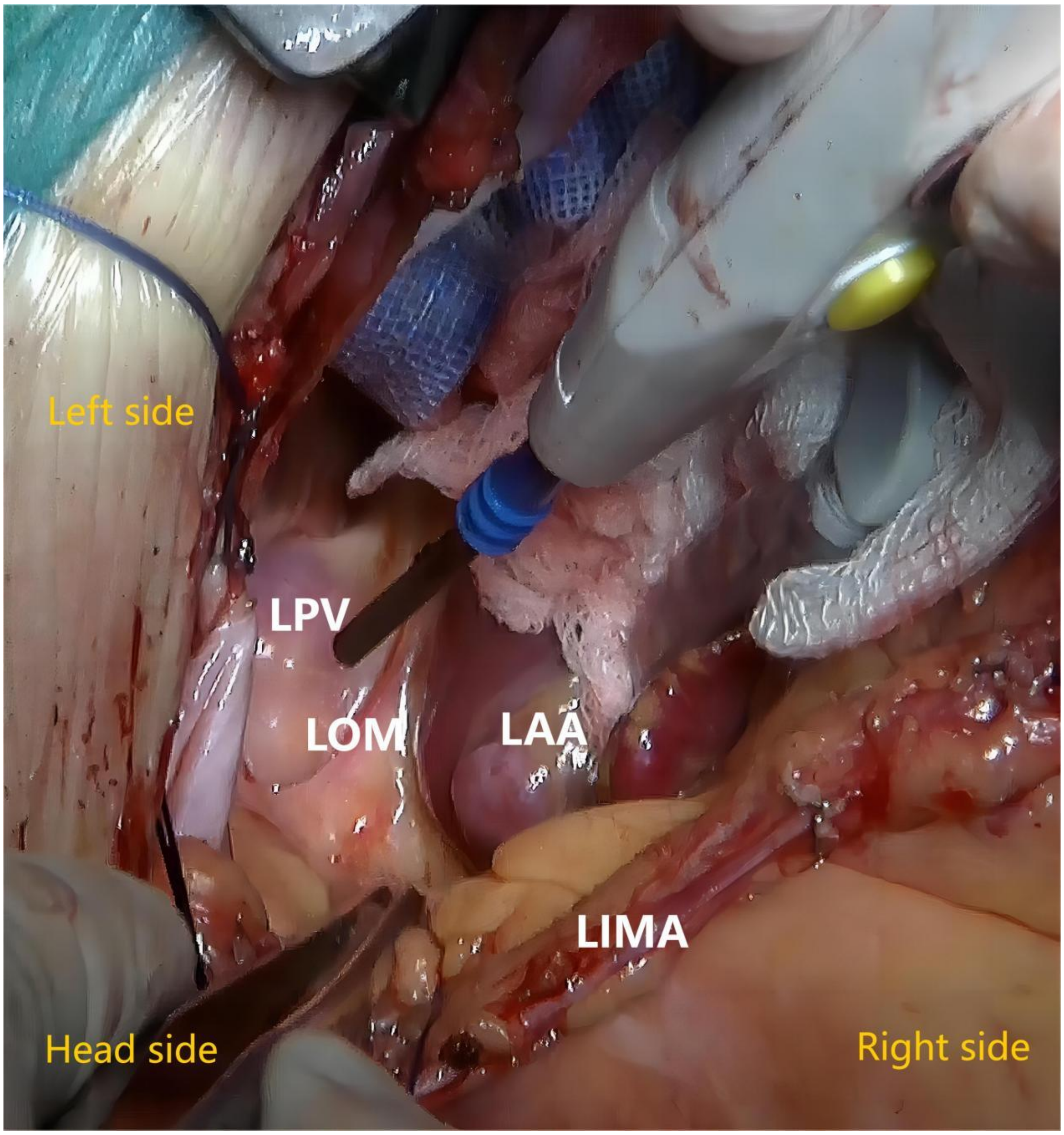

© 2024 Yang Z et al. *JAMA Cardiology.*

eFigure 2. Anatomic site of the fat pad along the Waterston groove. IVC, inferior vena cava; RAA, right atrial appendage; SVC, superior vena cava.

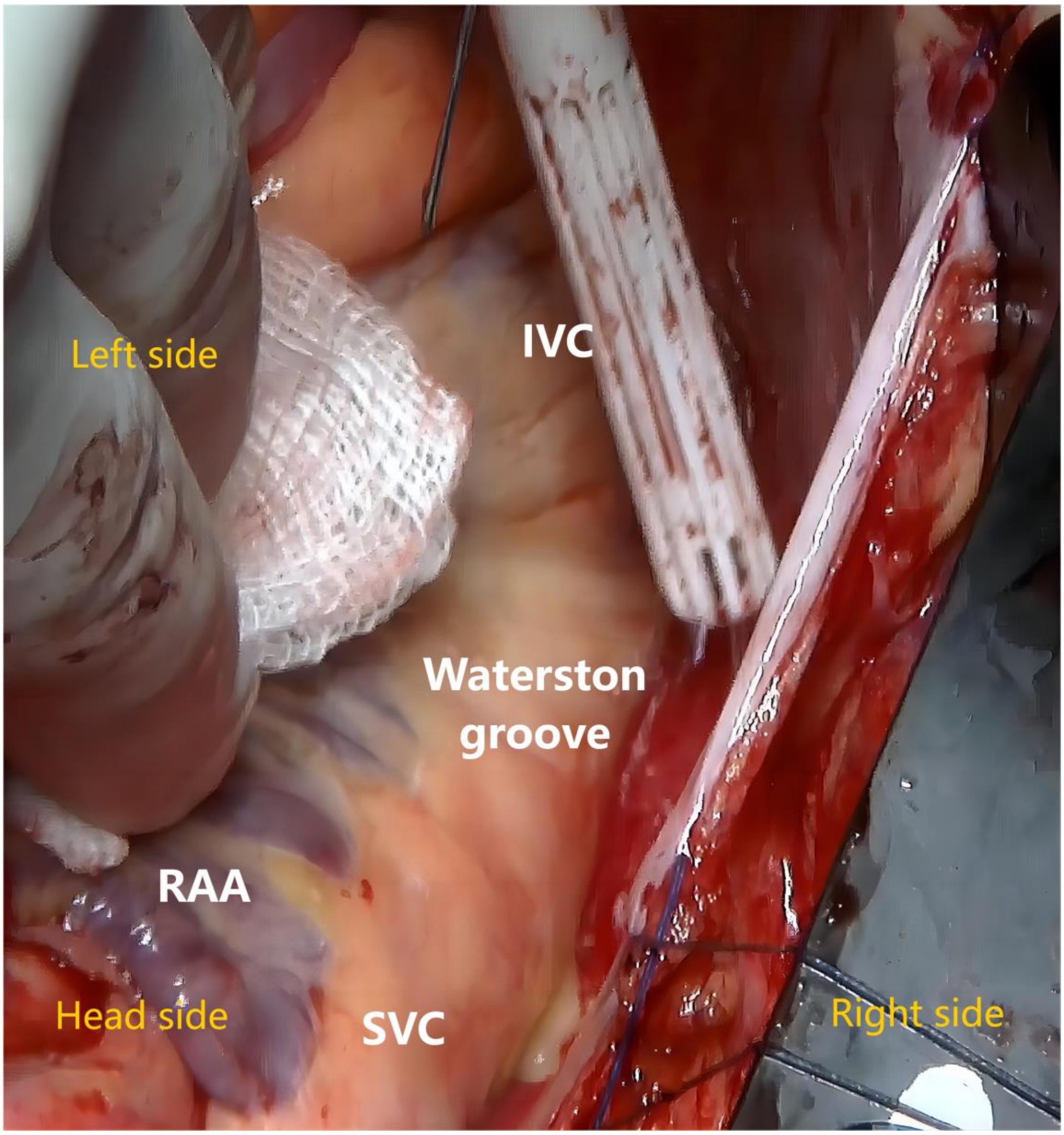

© 2024 Yang Z et al. *JAMA Cardiology.*

eFigure 3. Example of the fat pad along the Waterston groove. Figure A shows external (epicardial) side of sample. Figure B shows the internal (myocardial) side of the sample.

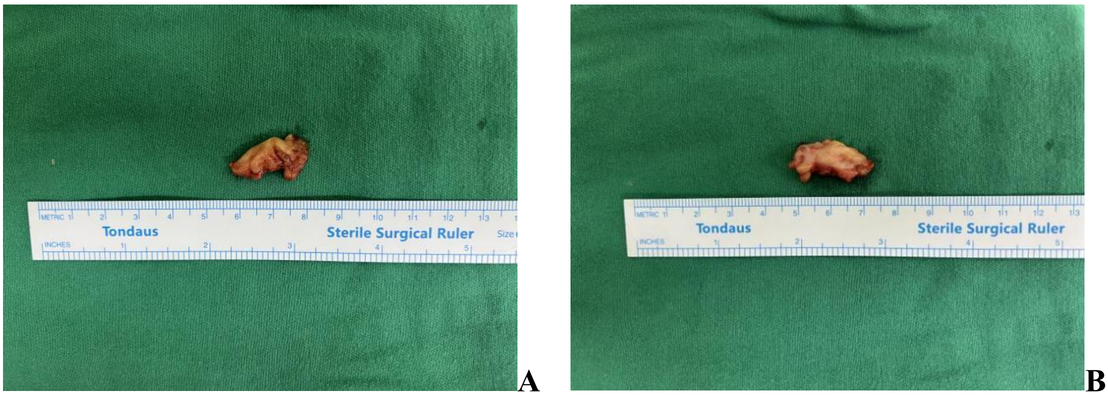

A B

© 2024 Yang Z et al. *JAMA Cardiology.*

eFigure 4. Examples of the histologic results performed on the fat pad along the Waterston groove. Figure A-B came from patient No.1 who developed POAF, a-e are nerve fibers and f-g are ganglia. Figure C-D came from patient No.11 who didn't develop POAF, h-k are nerve fibers.

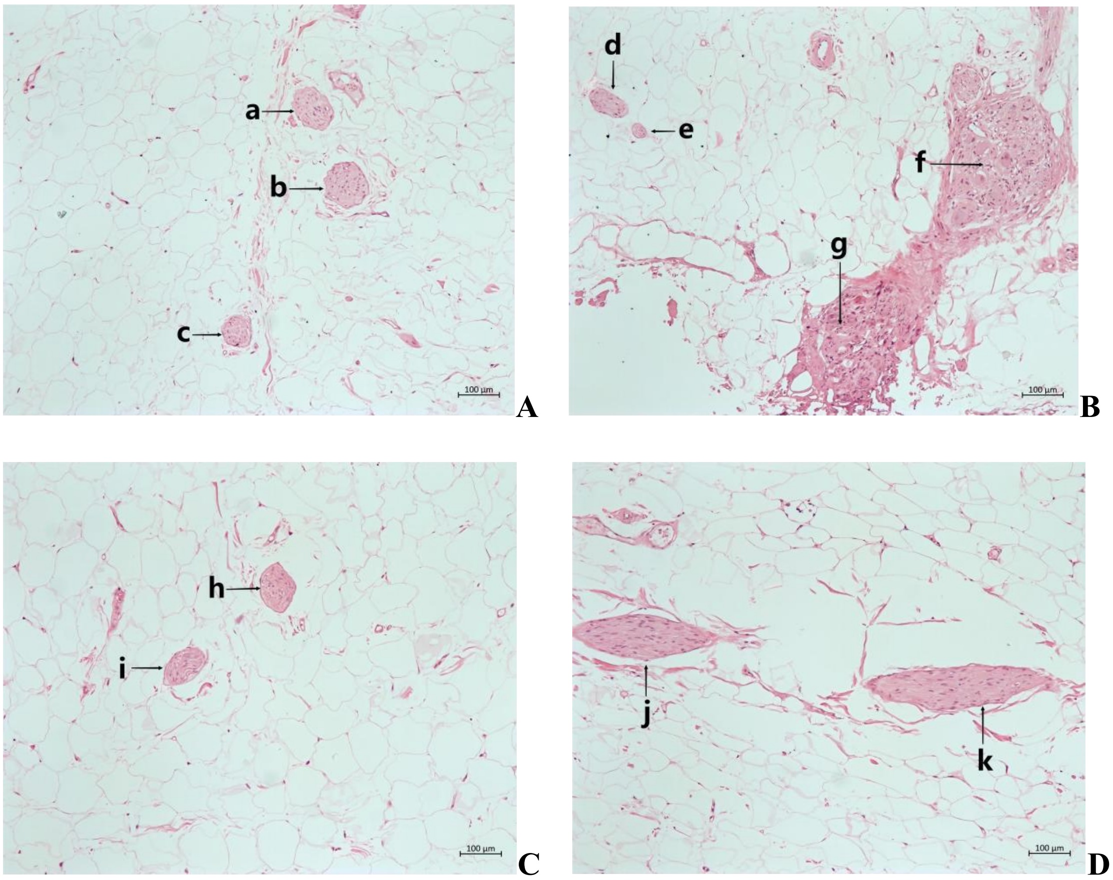

© 2024 Yang Z et al. *JAMA Cardiology.*

eFigure 5. Subgroup analysis for primary outcome. BMI, body mass index; CI, confidence interval; DM, diabetes mellitus; EF, ejection fraction; LA, left atrium; RR,risk ratio.

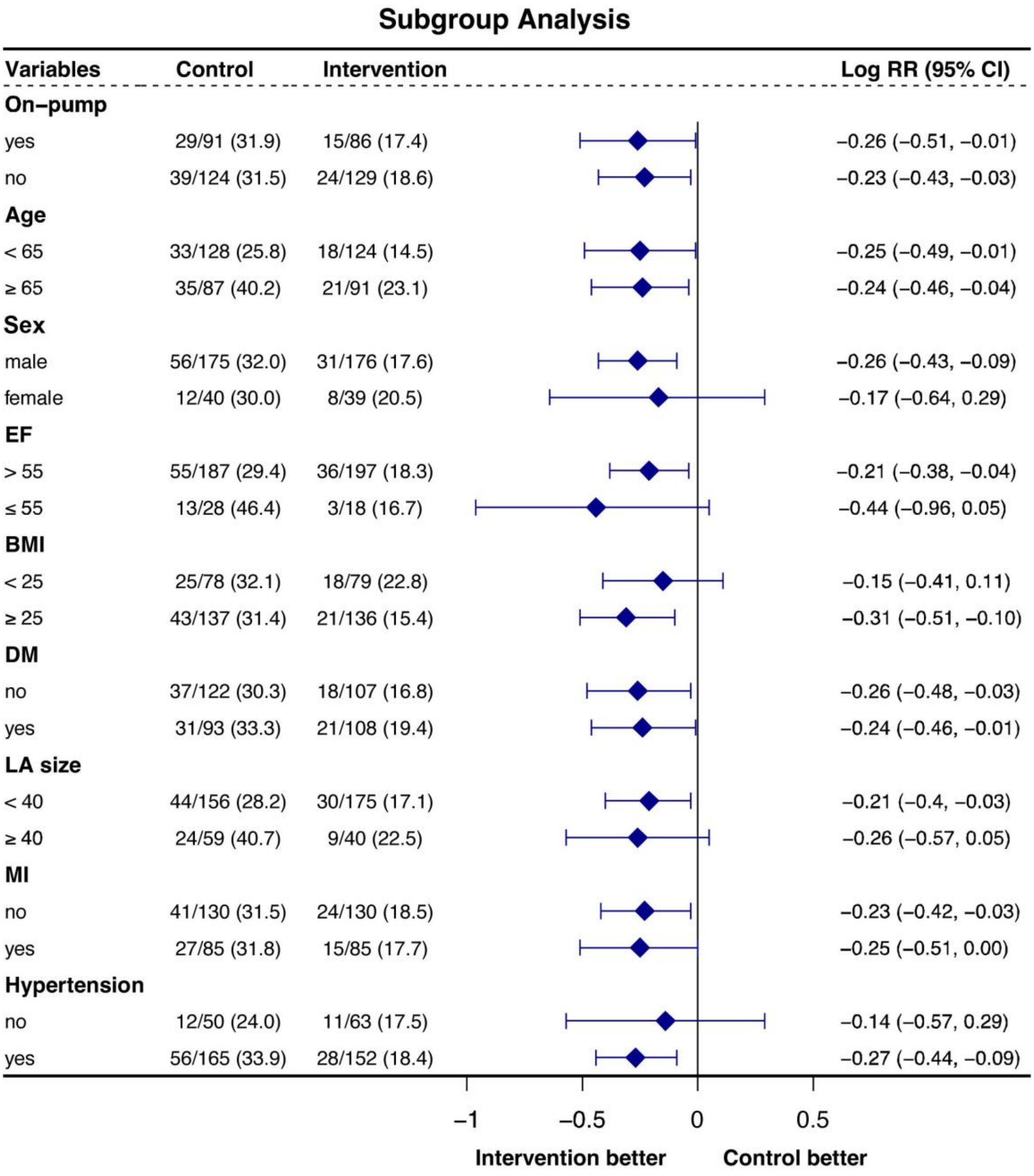

© 2024 Yang Z et al. *JAMA Cardiology.*
