# Supplementary material for: Partial Cardiac Denervation to Prevent Postoperative Atrial Fibrillation After Coronary Artery Bypass Grafting: The pCAD-POAF Randomized Clinical Trial
Source: JAMA Cardiol. 2024 Nov 17:e244639. Online ahead of print. doi: 10.1001/jamacardio.2024.4639 (PMC11571071; doi:10.1001/jamacardio.2024.4639)
Supplement: Supplement 2. — Trial Protocol and Statistical Analysis Plan [file jamacardiol-e244639-s002.pdf]

Partial CArdiac Denervation to Prevent PostOperative Atrial Fibrillation After Coronary Artery Bypass Grafting (pCAD-POAF)

Corresponding author: Wei Feng (fengwei@fuwai.com)

This supplement contains the following items:

1. Original protocol (pages 2-27)

2. Final protocol (pages 28-53)

3. Summary of changes (page 54-55)

4. Original statistical analysis plan (pages 56-73)

5. Final statistical analysis plan (pages 74-91)

6. Summary of changes (page 92)

Partial CArdiac Denervation to Prevent PostOperative Atrial Fibrillation After Coronary Artery Bypass Grafting

Principal Investigator: Wei Feng, MD, PhD, Director of Department of Cardiovascular Surgery， Fuwai Hospital, Beijing, China

Sponsor: Fuwai Hospital, National Center for Cardiovascular Diseases, National Clinical Research Center for Cardiovascular Diseases, Chinese Academy of Medical Sciences and Peking Union Medical College, Beijing, China.

Study Number: NCRC2020003
Version Number: 1.0
Date 05 December 2020

Table of Contents

Title page 2

Abbreviations 5

1.Introduction 6

1.1 Background 6

1.2 Research hypothesis 8

1.3 Rationale of conducting this trial 8

1.4 Benefit /risk and ethical assessment 9

2. Study objective 9

2.1 Primary objective 9

2.2 Secondary objectives 10

2.2.1 Safety assessments 10

2.2.2 Economic assessments 10

2.3 Exploratory objective 10

3.Study plan and procedures 11

3.1Overall study design and participants' timeline 11

3.2 Partial cardiac denervation procedure 13

4.Eligibility criteria 14

4.1 Inclusion criteria 15

4.2 Exclusion criteria 15

5. Study conduct 15

5.1 Patients enrollment 16

5.2 Randomization 16

5.3 Blinding 16

5.4 Concomitant care 17

5.5 Withdrawal from study 17

6.Data collection 17

6.1 Recording of data 17

6.2 Data collection at enrollment 18

6.3 Data collection at postoperative hospitalization 18

6.4 Data collection at follow-up visit 19

7.Adverse events 19

7.1 Definition of adverse event 20

7.2 Definition of serious adverse event 20

7.3 Recording of AEs and SAEs 20

7.4 Reporting of serious adverse events 21

8. Statistical analysis methods and sample size calculation 21

8.1 Statistical analysis methods 21

8.2 Sample size calculation 21

9. Data monitoring committee 23

References: 24

Abbreviations

AE, adverse event

AF, atrial fibrillation

ANMTs, autonomic neuromodulation therapies

CABG, coronary artery bypass grafting

CANS, cardiac autonomic nerve system

DMC, Data Monitoring Committee

ECG, electrocardiogram

GP, ganglionated plexi

LOM, ligament of Marshall

MACCE, major adverse cardiovascular and cerebrovascular events

POAF, postoperative atrial fibrillation

SAE, serious adverse event

1.Introduction

1.1 Background

Postoperative atrial fibrillation (POAF) is one of the major complications after cardiac surgery, which occurs mostly within one week after the operation, with an incidence of 10–50% [1, 2]. POAF has been proven to increase the risk of hemodynamic deterioration, heart failure and stroke, resulting in increased hospital-stay as well as the medical expenses [3]. While the incidence of POAF after coronary artery bypass grafting (CABG) ranges from 5% to 40% [4], it could result in severe postoperative circulatory fluctuations and expose patients to high risk of systemic embolism, such as stroke. Therefore, preventing POAF after CABG is essential.

The mechanism of POAF still remains unclear. Existing opinions are as follows: 1) disorder of cardiac autonomic nerve system (CANS), 2) inflammation, 3) oxidative stress, 4) abnormal activation of Ca^{+} channels, and 5) other potential mechanisms [5]. Among these possible mechanisms, the activation of CANS plays an important role in the occurrence of POAF. In fact, beta-blocker, which mainly inhibits cardiac sympathetic excitability, is now the only drug listed as Class I recommendation by the present guideline to prevent POAF [5]. However, previous study showed that even after administration of beta-blockers with a rate up to 80%, the incidence of POAF after CABG is as high as 21.1% [6].

Zafeiropoulos et al. [7] meticulously summarized the potential role of autonomic neuromodulation therapies (ANMTs) in preventing POAF after cardiac surgery. Studies also tried to reduce the incidence of POAF through surgical intervention of CANS during cardiac surgery, including resection of fat pads, ganglionated plexi (GP) ablation and botulinum toxin injection into epicardial fat pads [8-10]. However, these studies differed in several aspects, including population size, eligibility criteria, randomization approach, surgical procedure and non-optimal electrocardiogram (ECG) monitoring strategies. As a result, they failed to reveal the potential benefit of ANMTs. More recently, Wang et al. [11] reported a promising result of POAF reduction by Calcium Chloride injection into four major GPs during off-pump CABG (15% vs 36%, p=0.001), and the NeurOtoxin for the PreVention of Post-Operative Atrial Fibrillation trial also showed similar outcomes in isolated CABG patients, as well as in patients with advanced age receiving lower dose of botulinum toxin type A after cardiac surgery.

Partial cardiac denervation by resecting epicardial adipose tissue is also one of the ANMTs. Several previous studies [8, 12, 13] evaluated the efficacy of ventral cardiac denervation through resecting fat pads surrounding the great vessels of heart on the prevention of POAF. Unfortunately, however, the results were inconsistent due to the heterogeneity of enrolled population, limited sample size and incomplete monitoring timeline.

1.2 Research hypothesis

This study is designed to evaluate the efficacy of partial cardiac denervation, which will be achieved by cutting off the ligament of Marshall (LOM) and resecting the fat pad along the Waterston groove, on the prevention of POAF.

1.3 Rationale of conducting this trial

As mentioned before, there is lack of specific recommendation of approach for preventing POAF after CABG except of beta-blockers [5] and the related surgical means of ANMTs were still underestimated. Therefore, here in this study, we will focus on reevaluating the effect of partial cardiac denervation on preventing POAF after CABG in a larger population through more complete and longer duration of continuous ECG monitoring. To begin with, previous studies showed that the ligament of Marshall (LOM) is the critical site of CANS participating in the occurrence of atrial fibrillation (AF) [8]. Kim et al. [14] noticed that the LOM contained sympathetic nerve fibers, and had insertions into the myocardium of the left atrium and coronary sinus, providing the essentials for the formation of arrythmia. Other reports [15-17] clearly implicated the LOM and adjacent atrium as the origins of arrhythmias. In addition, Haemers et al. [18] showed that right atrium was obviously infiltrated by adipose tissue in patients with AF, especially in those with persistent AF. Besides, cutting off the LOM and resecting the fat pad along the Waterston groove is routinely used in maze surgery to treat patients with AF. Lastly, this trial also aims to seek for a safe, simple and convenient

way to prevent POAF after CABG. We believe that choosing one site at both right and left atrium namely the LOM and the fat pad along the Waterston groove, exactly meets our purpose.

1.4 Benefit /risk and ethical assessment

The intervention group will receive additional partial cardiac denervation procedure, namely cutting off the LOM and resecting the fat pad along the Waterston groove. Potential risks are as follows: extending operation time, increasing intraoperative blood loss and postoperative arrhythmias. The related technique has been applied in surgical treatment of AF and proved to be safe and feasible. The surgery removed only part of the CANS and will not affect the patient's normal sinus rhythm. All the participants can contact the study physician who will help them to get the right medical treatment, if any suspected arrhythmias-related adverse events emerge after surgery. The study will be approved by the Institutional Review Board of Fuwai Hospital.

2. Study objective

2.1 Primary objective

The primary outcome is POAF in 6 days, defined as a supraventricular arrhythmia lasting for >30 seconds [19]. Patients will be monitored continuously beginning within one hour after the surgery to the 6th day postoperatively through the NS-SP-B-01

Attached Dynamic ECG Recording System. In addition, the overall lasting time of supraventricular arrhythmia will also be recorded for evaluating AF burden. Heart rates will be continuously monitored, including lowest/highest/average heart beats, premature atrial/ventricular contractions etc. Two independent and blinded research doctors will interpret the ECG and determine the existence of POAF.

2.2 Secondary objectives

2.2.1 Safety assessments

1) The incidence of transferring to on-pump CABG intraoperatively;

2) The need for blood transfusion;

3) Re-operation for postoperative bleeding caused by partial cardiac denervation procedure;

4) The incidence of epicardial effusion within 30 days after discharge;

5) Arrhythmias other than AF within 30 days after discharge;

2.2.2 Economic assessments

6) Length of hospitalization;

7) All costs during hospitalization;

8) All costs after the surgery;

2.3 Exploratory objective

None.

3.Study plan and procedures

3.1Overall study design and participants’ timeline

Figure 1 shows the SPIRIT (Standard Protocol Items: Recommendations for Interventional Trials) guidelines [20] instructing the designation of our study.

	STUDY PERIOD				
	Enrolment	Allocation	Post-allocation		
TIMEPOINT	*Pre Intervention*	T_0	*Surgery*	*Post-operative Hospital stay*	*Follow-up Clinic Visit*
**ENROLMENT:**					
**Eligibility screen**	X				
**Informed consent**	X				
**Allocation**		X			
**INTERVENTIONS:**					
Control(no intervention)			X		
Cardiac denervation			X		
**ASSESSMENTS:**					
Post-operative atrial fibrillation				X	X
Time spent in atrial fibrillation				X	
Blood transfusion			X	X	
Transferring to on-pump CABG			X		
Re-operation				X	
Delayed pericardial effusion					X
Arrhythmias exclude of AF				X	X
Postoperative length of hospital stay				X	
All costs after surgery				X	
MACCE during the 30-day follow-up					X

Figure 1. Standard Protocol Items: Recommendations for Interventional Trials (SPIRIT). AF, atrial fibrillation.

During the primary screening, patients will be evaluated for the eligibility into the study, and an individual informed consent will be signed at the willingness of each patient. Then, participants will be randomly allocated to either intervention or control

group. After surgery, monitoring for the occurrence of AF will be lasted until the 6th day postoperatively among all of the patients. Meanwhile, complications such as pericardial effusion will also be assessed. On the day of discharge, participants will be investigated by 12-lead standard ECG and echocardiogram. During the 30-day follow-up, information such as the participants' overall health status, medication use, and whether they have experienced any kind of arrythmia or MACCEs, will be collected. The participants will also be investigated by ECG and echocardiogram again for further assessment (**Figure 2**).

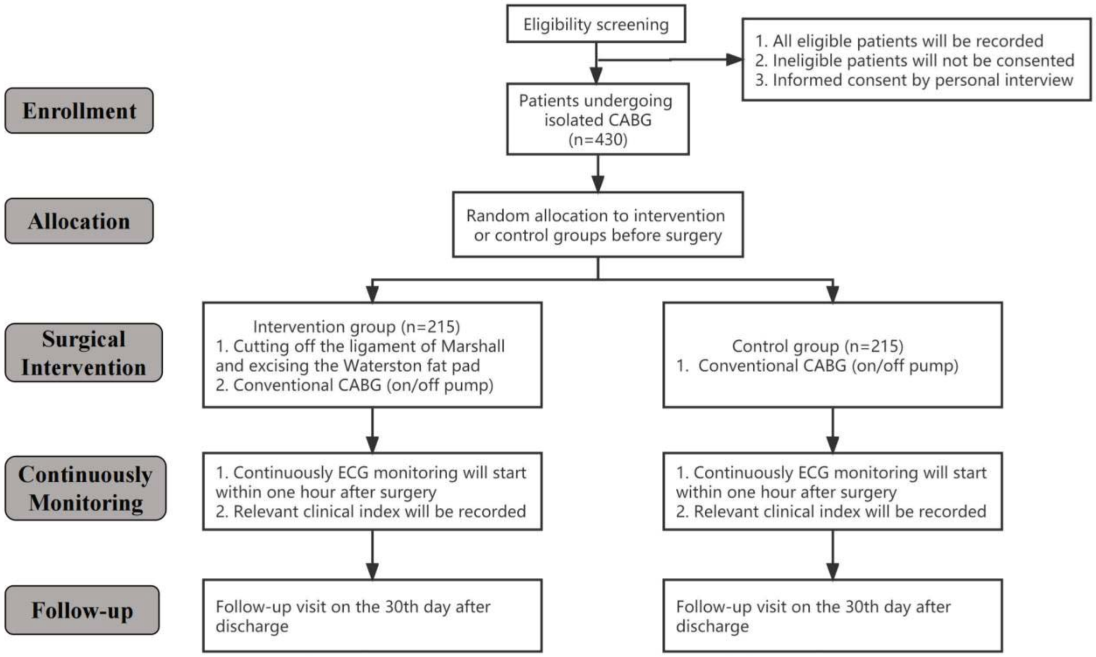

Figure 2. The study flow Chart. CABG, coronary artery bypass grafting; ECG, electrocardiogram.

3.2 Partial cardiac denervation procedure

In the intervention group, participant will undergo partial cardiac denervation through

cutting off the LOM and resecting the fat pad along the Waterston groove. Specifically, during the on-pump CABG, the heart will be pulled to the right to expose the LOM between the left atrial appendage and the left pulmonary veins under extracorporeal circulation, and the LOM will be cut off by electrotome. Then, the fat pad along the Waterston groove will be exposed between the right pulmonary veins and right atrium and excised completely to the surface of myocardium, with the upper edge extending beyond the opening of the right upper pulmonary vein and the lower edge to the inferior vena cava. For off-pump CABG, we will cut off the LOM in the same way mentioned above. Then if the patient's heart is well tolerated and hemodynamics is stable, a fixator can be used to fix the heart when resecting the fat pad along the Waterston groove. Otherwise, cardiac denervation will be performed after CABG is completed. The estimated additional surgery time is 5 minutes. In order to achieve a satisfactory efficacy, a thorough cardiac denervation to myocardium surface will be emphasized. In addition, the existence of ganglia will be further proved through histologic analysis using the fat pad samples collected from 10 pairs of patients with or without POAF, respectively.

4.Eligibility criteria

We will consecutively recruit adult patients who are undergoing CABG (on-pump/off-pump) and agree to participate in this clinical trial.

4.1 Inclusion criteria

1)Adult patients scheduled for isolated CABG.

2)Patients who are willing to participate in this study and sign the informed consent.

4.2 Exclusion criteria

1) age > 75;

2) emergent CABG;

3) history of cardiac surgery;

4) EF < 40%;

5) Moderate of severer mitral regurgitation;

6) simultaneously undergoing any other cardiac surgery;

7) critical condition requiring hemodynamic support before CABG, such as need for extracorporeal membrane oxygenation or intra-aortic balloon pump;

8) history of AF last 6 months;

9) receiving antiarrhythmic therapies except of taking beta-blockers last 2 weeks before surgery.

10) Untreated hypothyroidism.

11) Serum creatinine > 150 umol/L.

5. Study conduct

5.1 Patients enrollment

Patients who are ready for CABG and eligible for the study will be included after obtaining the informed consent form.

5.2 Randomization

A controlled, randomized process will be used to assign participants to treatment groups. A computer-generated minimized random allocation technique based on factors including age, sex, history of myocardial infarction, and left ventricular ejection fraction will be employed to ensure that the cases are distributed equally. The department research fellow will inform the surgeon of the patient's group assignment once they are under general anesthesia in the operating room.

5.3 Blinding

As different surgical operations are involved, complete double-blindness cannot be achieved. In order to reduce the researcher bias, surgeons are not allowed to know the result of patient’s assignment until surgery, and the third-party supervisor responsible for randomization shall inform the surgeons of the results only after general anesthesia and before the surgical procedure starts. Patients and data analysts will be blinded throughout the process. Unblinding will be done under the third-party supervision after all data analysis is completed.

5.4 Concomitant care

Following the present clinical practice, patients will continue on taking beta-blockers till the time of operation if they are already taking the medicine preoperatively. After conventional CABG, all of our patients will be prescribed with beta-blockers unless there is contraindication, such as complication with bradycardia (heart rate less than 65 beats per minute), atrioventricular block, or requiring epicardial pacing. For further evaluation of heart rhythm and function, patients will receive transthoracic echocardiogram and ECG before discharge and at the 30-day follow-up.

5.5 Withdrawal from study

Since the primary outcome will be obtained in patients' hospitalization and the follow-up visit will only be 30 days after discharge, we don't expect too many drop out or loss. A certain proportion of sample size will take the withdrawal rate into account.

6.Data collection

6.1 Recording of data

By filling out the specific Case Report Form, data collection will be completed in a prospective manner throughout the hospital-stay. Final data sets will be accessible to the primary investigator, statistician, and other researchers. One of the research doctors will have access to the database and will be required to ensure the accuracy

and quality of the data.

6.2 Data collection at enrollment

Adult patients scheduled for isolated CABG will be screened for eligibility. Patients who meet the eligibility criteria and willing to give written informed consent will be invited to participate. At this period, following baseline characteristics will be collected:

1) Age, sex, height, weight, medical history, family history and physical examination;

2) Comprehensive laboratory tests and examinations: blood, biochemistry, coagulation, biomarkers, transthoracic echocardiography, peripheral artery/vein ultrasound, chest radiography, ECG, entire aorta CT, lung function/chest CT/head CT/cardiac MR (if necessary);

3) Coronary artery evaluation according to coronary angiogram, including atherosclerotic sites and stenosis degree;

4) Other comorbidities and concomitant medications.

6.3 Data collection at postoperative hospitalization

Since POAF is influenced by a great deal of aspects, data collection shall be comprehensive. Both intraoperative characteristics and postoperative data will be collected at this period:

1)Operation time, cardiopulmonary time, cross clamp time, number of total/artery/venous grafts.

2)Postoperative laboratory tests and examinations: blood, biochemistry, coagulation, biomarker, transthoracic echocardiography and 12-lead-ECG at discharge.

3)Intensive care unit stay, length of hospitalization, postoperative costs, blood transfusion, secondary operation, complications such as stroke, acute kidney injury and postoperative myocardial infarction.

4)The prescription of medications in postoperative hospitalization and at discharge.

5)The continuous ECG monitoring device will be removed from participants at discharge and handed to function test department for data exportation and analysis.

6.4 Data collection at follow-up visit

The 30-day follow-up after discharge will be completed by outpatient visit and/or phone calls. During this period, following data will be collected:

1) MACCE: the composite of all-cause death, myocardial infarction, stroke and repeat coronary revascularization during these 30 days.

2)Transthoracic echocardiography and 12-lead-ECG at the 30 day after discharge.

3)Any arrhythmias experience confirmed by ECG and related treatment.

7.Adverse events

The Principal Investigator is responsible for ensuring that all staff involved in the study is familiar with the content of this section.

7.1 Definition of adverse event

The occurrence of an undesirable medical condition or the worsening of an already-existing medical condition after or during exposure to the designated intervention procedure is referred to as an adverse event (AE), regardless of whether it is thought to be directly related to the procedure or not. The unexpected medical condition can exhibit as symptoms, signs or the abnormal results of clinical tests, which may occur at any time after the patient decide to participate this trial. The term AE is used to include both serious and non-serious AEs.

7.2 Definition of serious adverse event

A serious adverse event (SAE) is an AE which meets one or more criteria as follows:

1)Leads to life-threatening condition.

2)Is the direct cause of death.

3)Causes complications needing sustained medical therapy.

4)Seriously worsens the life quality of participant.

Any SAE must be assessed and evaluated by investigators.

7.3 Recording of AEs and SAEs

All AEs and SAEs must be recorded in Case Report Form.

The following variables will be recorded for AE:

1)AE (verbatim);

2)The date when the AE started and stopped;

3)Whether the AE is serious or not;

4)Action taken with regard to investigational procedure;

5)AE caused subject's withdrawal from study (yes or no);

6)Outcome.

In addition, the following variables will be recorded for SAE:

1)Date AE develops to SAE;

2)The reasons of AE develop to SAE;

3)Date investigators be aware of SAE;

4)Date of hospitalization/discharge/death.

7.4 Reporting of serious adverse events

Investigators are responsible for meeting all regulatory reporting requirements reporting SAE to Ethics Committee in time. Investigators must inform the local authority of any SAE within 24 hours in accordance with the local regulations.

8. Statistical analysis methods and sample size calculation

8.1 Statistical analysis methods

Continuous variables will be tested by Student's t test if normally distributed; otherwise, by the Mann-Whitney U test. Chi-squared test or Fisher's exact test will be applied for categorical variables, as appropriate. The analysis of the primary outcomes will be carried out through an intention-to-treat approach. Meanwhile, as-treated,

per-protocol and/or modified intention-to-treat analysis will also be conducted. A sensitivity analysis regarding the primary outcome will be carried out by the time-to-event analysis performed with Kaplan-Meier survival curves and compared by the log-rank test. Meanwhile, subgroup analysis will be conducted based on the risk factors of POAF reported by previous studies, including sex, age (≥ 65 years vs. < 65 years), LVEF (> 55% vs. ≤ 55%), body mass index (≥ 25 kg/m^2 vs. < 25 kg/m^2), left atrium size (≥ 40 mm vs. < 40 mm), and history of myocardial infarction, diabetes mellitus and hypertension. Secondary outcomes including safety and economics will also be analyzed. Two tailed test will be applied, and a p-value < 0.05 will be considered statistically significant. Statistical analyses will be performed using R 4.0.2 (R Core Team, Vienna, Austria) and Stata 15.0 (StataCorp，College Station，TX，USA).

8.2 Sample size calculation

Two parallel arms will be required. The occurrence of POAF after cardiac surgery was about 23%, according to the prior study [19]. Based on a detailed reviewing of the existing studies, cardiac denervation reduces the incidence of POAF by 50% [21]. Therefore, with 80% power and 0.05 alpha, 408 participants are needed to detect 23% POAF rate in the control group and a reduction by 50% in the intervention group. Taking into account a 5% protocol violation rate and patient loss/dropout, a population size of 430 (215 in each group) shall be sufficient for this investigation. As one of the world’s largest centers of cardiovascular diseases, Fuwai Hospital

completes more than 10 thousand cardiac surgeries every year, of which an approximate of 5 thousand are isolated CABG. This trial shall be completed in due course.

9.Data monitoring committee

The Data Monitoring Committee (DMC) is composed of one cardiologist, one cardiac surgeon, one cardiac anesthesiologist, one bioethicist and one statistician who are not involved in this trial. The Institutional Review Board and the DMC will be awared once there are SAEs. If there is a significant difference of mortality or SAEs between the two groups and it is considered to be a direct result of this cardiac partial denervation approach, the study will be stopped.

References:

1. Ascione R, Caputo M, Calori G, Lloyd CT, Underwood MJ, Angelini GD. Predictors of atrial fibrillation after conventional and beating heart coronary surgery: A prospective, randomized study. *Circulation* 2000;102:1530-1535.

2. Bharucha DB, Kowey PR. Management and prevention of atrial fibrillation after cardiovascular surgery. *Am J Cardiol* 2000;85:20D-24D.

3. Aranki SF, Shaw DP, Adams DH, Rizzo RJ, Couper GS, VanderVliet M, Collins JJ, Cohn LH, Burstin HR. Predictors of atrial fibrillation after coronary artery surgery. Current trends and impact on hospital resources. *Circulation* 1996;94:390-397.

4. Katritsis DG, Pokushalov E, Romanov A, Giazitzoglou E, Siontis GC, Po SS, Camm AJ, Ioannidis JP. Autonomic denervation added to pulmonary vein isolation for paroxysmal atrial fibrillation: a randomized clinical trial. *J Am Coll Cardiol* 2013;62:2318-2325.

5. Frendl G, Sodickson AC, Chung MK, Waldo AL, Gersh BJ, Tisdale JE, Calkins H, Aranki S, Kaneko T, Cassivi S, Smith SJ, Darbar D, Wee JO, Waddell TK, Amar D, Adler D. 2014 AATS guidelines for the prevention and management of perioperative atrial fibrillation and flutter for thoracic surgical procedures. *J Thorac Cardiovasc Surg* 2014;148:e153-e193.

6. Zheng Z, Jayaram R, Jiang L, Emberson J, Zhao Y, Li Q, Du J, Guarguagli S, Hill M, Chen Z, Collins R, Casadei B. Perioperative Rosuvastatin in Cardiac Surgery. *N Engl J Med* 2016;374:1744-1753.

7. Zafeiropoulos S, Doundoulakis I, Farmakis IT, Miyara S, Giannis D, Giannakoulas G, Tsiachris D, Mitra R, Skipitaris NT, Mountantonakis SE, Stavrakis S, Zanos S. Autonomic Neuromodulation for Atrial Fibrillation Following Cardiac Surgery: JACC Review Topic of the Week. *J Am Coll Cardiol* 2022;79:682-694.

8. Melo J, Voigt P, Sonmez B, Ferreira M, Abecasis M, Rebocho M, Timoteo A, Aguiar C, Tansal S, Arbatli H, Dion R. Ventral cardiac denervation reduces the incidence of atrial fibrillation after coronary artery bypass grafting. *J Thorac Cardiovasc Surg* 2004;127:511-516.

9. Al-Atassi T, Toeg H, Malas T, Lam BK. Mapping and ablation of autonomic ganglia in prevention of postoperative atrial fibrillation in coronary surgery: MAAPPAFS atrial fibrillation randomized controlled pilot study. *Can J Cardiol* 2014;30:1202-1207.

10. Romanov A, Pokushalov E, Ponomarev D, Bayramova S, Shabanov V, Losik D, Stenin I, Elesin D, Mikheenko I, Strelnikov A, Sergeevichev D, Kozlov B, Po SS, Steinberg JS. Long-term suppression of atrial fibrillation by botulinum toxin injection into epicardial fat pads in patients undergoing cardiac surgery: Three-year follow-up of a randomized study. *Heart Rhythm* 2019;16:172-177.

11. Wang H, Zhang Y, Xin F, Jiang H, Tao D, Jin Y, He Y, Wang Q, Po SS. Calcium-Induced Autonomic Denervation in Patients With Post-Operative Atrial Fibrillation. *J Am Coll Cardiol* 2021;77:57-67.

12. Alex J, Guvendik L. Evaluation of ventral cardiac denervation as a prophylaxis against atrial fibrillation after coronary artery bypass grafting. *Ann Thorac Surg*

2005;79:517-520.

13. Omran AS, Karimi A, Ahmadi H, Yazdanifard P, Sheikh FM, Tazik M. Prophylactic ventral cardiac denervation: does it reduce incidence of atrial fibrillation after coronary artery bypass grafting? *J Thorac Cardiovasc Surg* 2010;140:1036-1039.

14. Kim DT, Lai AC, Hwang C, Fan L, Karagueuzian HS, Chen P, Fishbein MC. The ligament of Marshall: a structural analysis in human hearts with implications for atrial arrhythmias. *J Am Coll Cardiol* 2000;36:1324-1327.

15. Hwang C, Karagueuzian HS, Chen PS. Idiopathic paroxysmal atrial fibrillation induced by a focal discharge mechanism in the left superior pulmonary vein: possible roles of the ligament of Marshall. *J Cardiovasc Electrophysiol* 1999;10:636-648.

16. Katritsis D, Ioannidis JP, Anagnostopoulos CE, Sarris GE, Giazitzoglou E, Korovesis S, Camm AJ. Identification and catheter ablation of extracardiac and intracardiac components of ligament of Marshall tissue for treatment of paroxysmal atrial fibrillation. *J Cardiovasc Electrophysiol* 2001;12:750-758.

17. Polymeropoulos KP, Rodriguez LM, Timmermans C, Wellens HJ. Images in cardiovascular medicine. Radiofrequency ablation of a focal atrial tachycardia originating from the Marshall ligament as a trigger for atrial fibrillation. *Circulation* 2002;105:2112-2113.

18. Haemers P, Hamdi H, Guedj K, Suffee N, Farahmand P, Popovic N, Claus P, LePrince P, Nicoletti A, Jalife J, Wolke C, Lendeckel U, Jais P, Willems R, Hatem SN.

Atrial fibrillation is associated with the fibrotic remodelling of adipose tissue in the subepicardium of human and sheep atria. *Eur Heart J* 2017;38:53-61.

19. Abouarab AA, Leonard JR, Ohmes LB, Lau C, Rong LQ, Ivascu NS, Pryor KO, Munjal M, Crea F, Massetti M, Sanna T, Girardi LN, Gaudino M. Posterior Left pericardiotomy for the prevention of postoperative Atrial fibrillation after Cardiac Surgery (PALACS): study protocol for a randomized controlled trial. *Trials* 2017;18:593.

20. Chan AW, Tetzlaff JM, Gotzsche PC, Altman DG, Mann H, Berlin JA, Dickersin K, Hrobjartsson A, Schulz KF, Parulekar WR, Krleza-Jeric K, Laupacis A, Moher D. SPIRIT 2013 explanation and elaboration: guidance for protocols of clinical trials. *BMJ* 2013;346:e7586.

21. Biancari F, Mahar MA. Meta-analysis of randomized trials on the efficacy of posterior pericardiotomy in preventing atrial fibrillation after coronary artery bypass surgery. J Thorac Cardiovasc Surg 2010;139:1158-1161.

Partial CArdiac Denervation to Prevent PostOperative Atrial Fibrillation After Coronary Artery Bypass Grafting

Principal Investigator: Wei Feng, MD, PhD, Director of Department of Cardiovascular Surgery， Fuwai Hospital, Beijing, China

Sponsor: Fuwai Hospital, National Center for Cardiovascular Diseases, National Clinical Research Center for Cardiovascular Diseases, Chinese Academy of Medical Sciences and Peking Union Medical College, Beijing, China.

Study Number: NCRC2020003
Version Number: 2.0
Date 30 April 2022

Table of contents

Title page 28

Abbreviations 31

1.Introduction 32

1.1 Background 32

1.2 Research hypothesis 34

1.3 Rationale of conducting this trial 34

1.4 Benefit /risk and ethical assessment 35

2.Study objective 35

2.1 Primary objective 35

2.2 Secondary objectives 36

2.2.1 Safety assessments 36

2.2.2 Economic assessments 36

2.3 Exploratory objective 36

3.Study plan and procedures 37

3.1Overall study design and participants' timeline 37

3.2 Partial cardiac denervation procedure 39

4.Eligibility criteria 40

4.1 Inclusion criteria 41

4.2 Exclusion criteria 41

5. Study conduct 41

5.1 Patients enrollment 41

5.2 Randomization 42

5.3 Blinding 42

5.4 Concomitant care 42

5.5 Withdrawal from study 43

6.Data collection 43

6.1 Recording of data 43

6.2 Data collection at enrollment 43

6.3 Data collection at postoperative hospitalization 44

6.4 Data collection at follow-up visit 45

7.Adverse events 45

7.1 Definition of adverse event 45

7.2 Definition of serious adverse event 46

7.3 Recording of AEs and SAEs 46

7.4 Reporting of serious adverse events 47

8. Statistical analysis methods and sample size calculation 47

8.1 Statistical analysis methods 47

8.2 Sample size calculation 47

9. Data monitoring committee 49

References: 50

Abbreviations

AE, adverse event

AF, atrial fibrillation

ANMTs, autonomic neuromodulation therapies

CABG, coronary artery bypass grafting

CANS, cardiac autonomic nerve system

DMC, Data Monitoring Committee

ECG, electrocardiogram

GP, ganglionated plexi

LOM, ligament of Marshall

MACCE, major adverse cardiovascular and cerebrovascular events

POAF, postoperative atrial fibrillation

SAE, serious adverse event

1.Introduction

1.1 Background

Postoperative atrial fibrillation (POAF) is one of the major complications after cardiac surgery, which occurs mostly within one week after the operation, with an incidence of 10–50% [1, 2]. POAF has been proven to increase the risk of hemodynamic deterioration, heart failure and stroke, resulting in increased hospital-stay as well as the medical expenses [3]. While the incidence of POAF after coronary artery bypass grafting (CABG) ranges from 5% to 40% [4], it could result in severe postoperative circulatory fluctuations and expose patients to high risk of systemic embolism, such as stroke. Therefore, preventing POAF after CABG is essential.

The mechanism of POAF still remains unclear. Existing opinions are as follows: 1) disorder of cardiac autonomic nerve system (CANS), 2) inflammation, 3) oxidative stress, 4) abnormal activation of Ca^{+} channels, and 5) other potential mechanisms [5]. Among these possible mechanisms, the activation of CANS plays an important role in the occurrence of POAF. In fact, beta-blocker, which mainly inhibits cardiac sympathetic excitability, is now the only drug listed as Class I recommendation by the present guideline to prevent POAF [5]. However, previous study showed that even after administration of beta-blockers with a rate up to 80%, the incidence of POAF after CABG is as high as 21.1% [6].

Zafeiropoulos et al. [7] meticulously summarized the potential role of autonomic neuromodulation therapies (ANMTs) in preventing POAF after cardiac surgery. Studies also tried to reduce the incidence of POAF through surgical intervention of CANS during cardiac surgery, including resection of fat pads, ganglionated plexi (GP) ablation and botulinum toxin injection into epicardial fat pads [8-10]. However, these studies differed in several aspects, including population size, eligibility criteria, randomization approach, surgical procedure and non-optimal electrocardiogram (ECG) monitoring strategies. As a result, they failed to reveal the potential benefit of ANMTs. More recently, Wang et al. [11] reported a promising result of POAF reduction by Calcium Chloride injection into four major GPs during off-pump CABG (15% vs 36%, p=0.001), and the NeurOtoxin for the PreVention of Post-Operative Atrial Fibrillation trial also showed similar outcomes in isolated CABG patients, as well as in patients with advanced age receiving lower dose of botulinum toxin type A after cardiac surgery.

Partial cardiac denervation by resecting epicardial adipose tissue is also one of the ANMTs. Several previous studies [8, 12, 13] evaluated the efficacy of ventral cardiac denervation through resecting fat pads surrounding the great vessels of heart on the prevention of POAF. Unfortunately, however, the results were inconsistent due to the heterogeneity of enrolled population, limited sample size and incomplete monitoring timeline.

1.2 Research hypothesis

This study is designed to evaluate the efficacy of partial cardiac denervation, which will be achieved by cutting off the ligament of Marshall (LOM) and resecting the fat pad along the Waterston groove, on the prevention of POAF.

1.3 Rationale of conducting this trial

As mentioned before, there is lack of specific recommendation of approach for preventing POAF after CABG except of beta-blockers [5] and the related surgical means of ANMTs were still underestimated. Therefore, here in this study, we will focus on reevaluating the effect of partial cardiac denervation on preventing POAF after CABG in a larger population through more complete and longer duration of continuous ECG monitoring. To begin with, previous studies showed that the ligament of Marshall (LOM) is the critical site of CANS participating in the occurrence of atrial fibrillation (AF) [8]. Kim et al. [14] noticed that the LOM contained sympathetic nerve fibers, and had insertions into the myocardium of the left atrium and coronary sinus, providing the essentials for the formation of arrythmia. Other reports [15-17] clearly implicated the LOM and adjacent atrium as the origins of arrhythmias. In addition, Haemers et al. [18] showed that right atrium was obviously infiltrated by adipose tissue in patients with AF, especially in those with persistent AF. Besides, cutting off the LOM and resecting the fat pad along the Waterston groove is routinely used in maze surgery to treat patients with AF. Lastly, this trial also aims to seek for a safe, simple and convenient

way to prevent POAF after CABG. We believe that choosing one site at both right and left atrium namely the LOM and the fat pad along the Waterston groove, exactly meets our purpose.

1.4 Benefit /risk and ethical assessment

The intervention group will receive additional partial cardiac denervation procedure, namely cutting off the LOM and resecting the fat pad along the Waterston groove. Potential risks are as follows: extending operation time, increasing intraoperative blood loss and postoperative arrhythmias. The related technique has been applied in surgical treatment of AF and proved to be safe and feasible. The surgery removed only part of the CANS and will not affect the patient's normal sinus rhythm. All the participants can contact the study physician who will help them to get the right medical treatment, if any suspected arrhythmias-related adverse events emerge after surgery. The study will be approved by the Institutional Review Board of Fuwai Hospital.

2.Study objective

2.1 Primary objective

The primary outcome is POAF in 6 days, defined as a supraventricular arrhythmia lasting for >30 seconds [19]. Patients will be monitored continuously beginning within one hour after the surgery to the 6th day postoperatively through the NS-SP-B-01

Attached Dynamic ECG Recording System. In addition, the overall lasting time of supraventricular arrhythmia will also be recorded for evaluating AF burden. Heart rates will be continuously monitored, including lowest/highest/average heart beats, premature atrial/ventricular contractions etc. Two independent and blinded research doctors will interpret the ECG and determine the existence of POAF.

2.2 Secondary objectives

2.2.1 Safety assessments

1) The incidence of transferring to on-pump CABG intraoperatively;

2) The need for blood transfusion;

3) Re-operation for postoperative bleeding caused by partial cardiac denervation procedure;

4) The incidence of epicardial effusion within 30 days after discharge;

5) Arrhythmias other than AF within 30 days after discharge;

2.2.2 Economic assessments

6) Length of hospitalization;

7) All costs during hospitalization;

8) All costs after the surgery;

2.3 Exploratory objective

None.

3. Study plan and procedures

3.1 Overall study design and participants' timeline

Figure 1 shows the SPIRIT (Standard Protocol Items: Recommendations for Interventional Trials) guidelines [20] instructing the designation of our study.

	STUDY PERIOD				
	Enrolment	Allocation	Post-allocation		
TIMEPOINT	*Pre Intervention*	T_0	*Surgery*	*Post-operative Hospital stay*	*Follow-up Clinic Visit*
**ENROLMENT:**					
**Eligibility screen**	X				
**Informed consent**	X				
**Allocation**		X			
**INTERVENTIONS:**					
Control(no intervention)			X		
Cardiac denervation			X		
**ASSESSMENTS:**					
Post-operative atrial fibrillation				X	X
Time spent in atrial fibrillation				X	
Blood transfusion			X	X	
Transferring to on-pump CABG			X		
Re-operation				X	
Delayed pericardial effusion					X
Arrhythmias exclude of AF				X	X
Postoperative length of hospital stay				X	
All costs after surgery				X	
MACCE during the 30-day follow-up					X

Figure 1. Standard Protocol Items: Recommendations for Interventional Trials (SPIRIT). AF, atrial fibrillation.

During the primary screening, patients will be evaluated for the eligibility into the study, and an individual informed consent will be signed at the willingness of each patient. Then, participants will be randomly allocated to either intervention or control

group. After surgery, monitoring for the occurrence of AF will be lasted until the 6th day postoperatively among all of the patients. Meanwhile, complications such as pericardial effusion will also be assessed. On the day of discharge, participants will be investigated by 12-lead standard ECG and echocardiogram. During the 30-day follow-up, information such as the participants' overall health status, medication use, and whether they have experienced any kind of arrythmia or MACCEs, will be collected. The participants will also be investigated by ECG and echocardiogram again for further assessment (**Figure 2**).

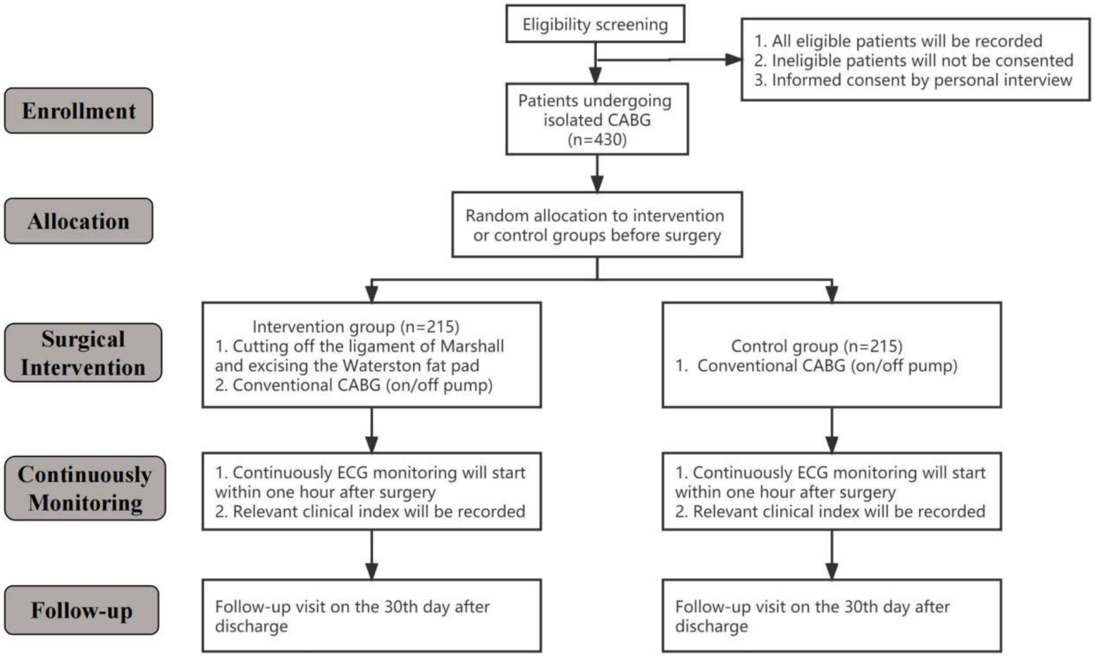

Figure 2. The study flow Chart. CABG, coronary artery bypass grafting; ECG, electrocardiogram.

3.2 Partial cardiac denervation procedure

In the intervention group, participant will undergo partial cardiac denervation through

cutting off the LOM and resecting the fat pad along the Waterston groove. Specifically, during the on-pump CABG, the heart will be pulled to the right to expose the LOM between the left atrial appendage and the left pulmonary veins under extracorporeal circulation, and the LOM will be cut off by electrotome. Then, the fat pad along the Waterston groove will be exposed between the right pulmonary veins and right atrium and excised completely to the surface of myocardium, with the upper edge extending beyond the opening of the right upper pulmonary vein and the lower edge to the inferior vena cava. For off-pump CABG, we will cut off the LOM in the same way mentioned above. Then if the patient's heart is well tolerated and hemodynamics is stable, a fixator can be used to fix the heart when resecting the fat pad along the Waterston groove. Otherwise, cardiac denervation will be performed after CABG is completed. The estimated additional surgery time is 5 minutes. In order to achieve a satisfactory efficacy, a thorough cardiac denervation to myocardium surface will be emphasized. In addition, the existence of ganglia will be further proved through histologic analysis using the fat pad samples collected from 10 pairs of patients with or without POAF, respectively.

4.Eligibility criteria

We will consecutively recruit adult patients who are undergoing CABG (on-pump/off-pump) and agree to participate in this clinical trial.

4.1 Inclusion criteria

1)Adult patients scheduled for isolated CABG.

2)Patients who are willing to participate in this study and sign the informed consent.

4.2 Exclusion criteria

1) age < 18;

2) emergent CABG;

3) history of cardiac surgery;

4) simultaneously undergoing any other cardiac surgery (such as Morrow procedure, valvular surgery, ventricular reconstruction due to ventricular aneurysm, or repair of the congenital heart diseases);

5) critical condition requiring hemodynamic support before CABG, such as need for extracorporeal membrane oxygenation or intra-aortic balloon pump;

6) history of AF;

7) receiving antiarrhythmic therapies except of taking beta-blockers last 2 weeks before surgery.

5. Study conduct

5.1 Patients enrollment

Patients who are ready for CABG and eligible for the study will be included after obtaining the informed consent form.

5.2 Randomization

A controlled, randomized process will be used to assign participants to treatment groups. A computer-generated minimized random allocation technique based on factors including age, sex, history of myocardial infarction, and left ventricular ejection fraction will be employed to ensure that the cases are distributed equally. The department research fellow will inform the surgeon of the patient's group assignment once they are under general anesthesia in the operating room.

5.3 Blinding

As different surgical operations are involved, complete double-blindness cannot be achieved. In order to reduce the researcher bias, surgeons are not allowed to know the result of patient’s assignment until surgery, and the third-party supervisor responsible for randomization shall inform the surgeons of the results only after general anesthesia and before the surgical procedure starts. Patients and data analysts will be blinded throughout the process. Unblinding will be done under the third-party supervision after all data analysis is completed.

5.4 Concomitant care

Following the present clinical practice, patients will continue on taking beta-blockers till the time of operation if they are already taking the medicine preoperatively. After conventional CABG, all of our patients will be prescribed with beta-blockers unless there is contraindication, such as complication with bradycardia (heart rate less than

65 beats per minute), atrioventricular block, or requiring epicardial pacing. For further evaluation of heart rhythm and function, patients will receive transthoracic echocardiogram and ECG before discharge and at the 30-day follow-up.

5.5 Withdrawal from study

Since the primary outcome will be obtained in patients' hospitalization and the follow-up visit will only be 30 days after discharge, we don't expect too many drop out or loss. A certain proportion of sample size will take the withdrawal rate into account.

6.Data collection

6.1 Recording of data

By filling out the specific Case Report Form, data collection will be completed in a prospective manner throughout the hospital-stay. Final data sets will be accessible to the primary investigator, statistician, and other researchers. One of the research doctors will have access to the database and will be required to ensure the accuracy and quality of the data.

6.2 Data collection at enrollment

Adult patients scheduled for isolated CABG will be screened for eligibility. Patients who meet the eligibility criteria and willing to give written informed consent will be

invited to participate. At this period, following baseline characteristics will be collected:

1) Age, sex, height, weight, medical history, family history and physical examination;

2) Comprehensive laboratory tests and examinations: blood, biochemistry, coagulation, biomarkers, transthoracic echocardiography, peripheral artery/vein ultrasound, chest radiography, ECG, entire aorta CT, lung function/chest CT/head CT/cardiac MR (if necessary);

3) Coronary artery evaluation according to coronary angiogram, including atherosclerotic sites and stenosis degree;

4) Other comorbidities and concomitant medications.

6.3 Data collection at postoperative hospitalization

Since POAF is influenced by a great deal of aspects, data collection shall be comprehensive. Both intraoperative characteristics and postoperative data will be collected at this period:

1) Operation time, cardiopulmonary time, cross clamp time, number of total/artery/venous grafts.

2) Postoperative laboratory tests and examinations: blood, biochemistry, coagulation, biomarker, transthoracic echocardiography and 12-lead-ECG at discharge.

3) Intensive care unit stay, length of hospitalization, postoperative costs, blood transfusion, secondary operation, complications such as stroke, acute kidney injury and postoperative myocardial infarction.

4) The prescription of medications in postoperative hospitalization and at discharge.

5) The continuous ECG monitoring device will be removed from participants at discharge and handed to function test department for data exportation and analysis.

6.4 Data collection at follow-up visit

The 30-day follow-up after discharge will be completed by outpatient visit and/or phone calls. During this period, following data will be collected:

1) MACCE: the composite of all-cause death, myocardial infarction, stroke and repeat coronary revascularization during these 30 days.

2) Transthoracic echocardiography and 12-lead-ECG at the 30 day after discharge.

3) Any arrhythmias experience confirmed by ECG and related treatment.

7.Adverse events

The Principal Investigator is responsible for ensuring that all staff involved in the study is familiar with the content of this section.

7.1 Definition of adverse event

The occurrence of an undesirable medical condition or the worsening of an already-existing medical condition after or during exposure to the designated intervention procedure is referred to as an adverse event (AE), regardless of whether it is thought to be directly related to the procedure or not. The unexpected medical

condition can exhibit as symptoms, signs or the abnormal results of clinical tests, which may occur at any time after the patient decide to participate this trial. The term AE is used to include both serious and non-serious AEs.

7.2 Definition of serious adverse event

A serious adverse event (SAE) is an AE which meets one or more criteria as follows:

1) Leads to life-threatening condition.
2) Is the direct cause of death.
3) Causes complications needing sustained medical therapy.
4) Seriously worsens the life quality of participant.

Any SAE must be assessed and evaluated by investigators.

7.3 Recording of AEs and SAEs

All AEs and SAEs must be recorded in Case Report Form.

The following variables will be recorded for AE:

1) AE (verbatim);
2) The date when the AE started and stopped;
3) Whether the AE is serious or not;
4) Action taken with regard to investigational procedure;
5) AE caused subject’s withdrawal from study (yes or no);
6) Outcome.

In addition, the following variables will be recorded for SAE:

1) Date AE develops to SAE;

2) The reasons of AE develop to SAE;

3) Date investigators be aware of SAE;

4) Date of hospitalization/discharge/death.

7.4 Reporting of serious adverse events

Investigators are responsible for meeting all regulatory reporting requirements reporting SAE to Ethics Committee in time. Investigators must inform the local authority of any SAE within 24 hours in accordance with the local regulations.

8. Statistical analysis methods and sample size calculation

8.1 Statistical analysis methods

Continuous variables will be tested by Student's t test if normally distributed; otherwise, by the Mann-Whitney U test. Chi-squared test or Fisher's exact test will be applied for categorical variables, as appropriate. The analysis of the primary outcomes will be carried out through an intention-to-treat approach. Meanwhile, as-treated, per-protocol and/or modified intention-to-treat analysis will also be conducted. A sensitivity analysis regarding the primary outcome will be carried out by the time-to-event analysis performed with Kaplan-Meier survival curves and compared by the log-rank test. Meanwhile, subgroup analysis will be conducted based on the risk factors of POAF reported by previous studies, including sex, age (≥ 65 years vs. <

65 years), LVEF (> 55% vs. ≤ 55%), body mass index (≥ 25 kg/m^2 vs. < 25 kg/m^2), left atrium size (≥ 40 mm vs. < 40 mm), and history of myocardial infarction, diabetes mellitus and hypertension. Secondary outcomes including safety and economics will also be analyzed. Two tailed test will be applied, and a p-value < 0.05 will be considered statistically significant. Statistical analyses will be performed using R 4.0.2 (R Core Team, Vienna, Austria) and Stata 15.0 (StataCorp，College Station，TX，USA).

8.2 Sample size calculation

Two parallel arms will be required. The occurrence of POAF after cardiac surgery was about 23%, according to the prior study [19]. Based on a detailed reviewing of the existing studies, cardiac denervation reduces the incidence of POAF by 50% [21]. Therefore, with 80% power and 0.05 alpha, 408 participants are needed to detect 23% POAF rate in the control group and a reduction by 50% in the intervention group. Taking into account a 5% protocol violation rate and patient loss/dropout, a population size of 430 (215 in each group) shall be sufficient for this investigation. As one of the world's largest centers of cardiovascular diseases, Fuwai Hospital completes more than 10 thousand cardiac surgeries every year, of which an approximate of 5 thousand are isolated CABG. This trial shall be completed in due course.

9. Data monitoring committee

The Data Monitoring Committee (DMC) is composed of one cardiologist, one cardiac surgeon, one cardiac anesthesiologist, one bioethicist and one statistician who are not involved in this trial. The Institutional Review Board and the DMC will be awared once there are SAEs. If there is a significant difference of mortality or SAEs between the two groups and it is considered to be a direct result of this cardiac partial denervation approach, the study will be stopped.

References:

1. Ascione R, Caputo M, Calori G, Lloyd CT, Underwood MJ, Angelini GD. Predictors of atrial fibrillation after conventional and beating heart coronary surgery: A prospective, randomized study. *Circulation* 2000;102:1530-1535.

2. Bharucha DB, Kowey PR. Management and prevention of atrial fibrillation after cardiovascular surgery. *Am J Cardiol* 2000;85:20D-24D.

3. Aranki SF, Shaw DP, Adams DH, Rizzo RJ, Couper GS, VanderVliet M, Collins JJ, Cohn LH, Burstin HR. Predictors of atrial fibrillation after coronary artery surgery. Current trends and impact on hospital resources. *Circulation* 1996;94:390-397.

4. Katritsis DG, Pokushalov E, Romanov A, Giazitzoglou E, Siontis GC, Po SS, Camm AJ, Ioannidis JP. Autonomic denervation added to pulmonary vein isolation for paroxysmal atrial fibrillation: a randomized clinical trial. *J Am Coll Cardiol* 2013;62:2318-2325.

5. Frendl G, Sodickson AC, Chung MK, Waldo AL, Gersh BJ, Tisdale JE, Calkins H, Aranki S, Kaneko T, Cassivi S, Smith SJ, Darbar D, Wee JO, Waddell TK, Amar D, Adler D. 2014 AATS guidelines for the prevention and management of perioperative atrial fibrillation and flutter for thoracic surgical procedures. *J Thorac Cardiovasc Surg* 2014;148:e153-e193.

6. Zheng Z, Jayaram R, Jiang L, Emberson J, Zhao Y, Li Q, Du J, Guarguagli S, Hill M, Chen Z, Collins R, Casadei B. Perioperative Rosuvastatin in Cardiac Surgery. *N Engl J Med* 2016;374:1744-1753.

7. Zafeiropoulos S, Doundoulakis I, Farmakis IT, Miyara S, Giannis D, Giannakoulas G, Tsiachris D, Mitra R, Skipitaris NT, Mountantonakis SE, Stavrakis S, Zanos S. Autonomic Neuromodulation for Atrial Fibrillation Following Cardiac Surgery: JACC Review Topic of the Week. *J Am Coll Cardiol* 2022;79:682-694.

8. Melo J, Voigt P, Sonmez B, Ferreira M, Abecasis M, Rebocho M, Timoteo A, Aguiar C, Tansal S, Arbatli H, Dion R. Ventral cardiac denervation reduces the incidence of atrial fibrillation after coronary artery bypass grafting. *J Thorac Cardiovasc Surg* 2004;127:511-516.

9. Al-Atassi T, Toeg H, Malas T, Lam BK. Mapping and ablation of autonomic ganglia in prevention of postoperative atrial fibrillation in coronary surgery: MAAPPAFS atrial fibrillation randomized controlled pilot study. *Can J Cardiol* 2014;30:1202-1207.

10. Romanov A, Pokushalov E, Ponomarev D, Bayramova S, Shabanov V, Losik D, Stenin I, Elesin D, Mikheenko I, Strelnikov A, Sergeevichev D, Kozlov B, Po SS, Steinberg JS. Long-term suppression of atrial fibrillation by botulinum toxin injection into epicardial fat pads in patients undergoing cardiac surgery: Three-year follow-up of a randomized study. *Heart Rhythm* 2019;16:172-177.

11. Wang H, Zhang Y, Xin F, Jiang H, Tao D, Jin Y, He Y, Wang Q, Po SS. Calcium-Induced Autonomic Denervation in Patients With Post-Operative Atrial Fibrillation. *J Am Coll Cardiol* 2021;77:57-67.

12. Alex J, Guvendik L. Evaluation of ventral cardiac denervation as a prophylaxis against atrial fibrillation after coronary artery bypass grafting. *Ann Thorac Surg*

2005;79:517-520.

13. Omran AS, Karimi A, Ahmadi H, Yazdanifard P, Sheikh FM, Tazik M. Prophylactic ventral cardiac denervation: does it reduce incidence of atrial fibrillation after coronary artery bypass grafting? *J Thorac Cardiovasc Surg* 2010;140:1036-1039.

14. Kim DT, Lai AC, Hwang C, Fan L, Karagueuzian HS, Chen P, Fishbein MC. The ligament of Marshall: a structural analysis in human hearts with implications for atrial arrhythmias. *J Am Coll Cardiol* 2000;36:1324-1327.

15. Hwang C, Karagueuzian HS, Chen PS. Idiopathic paroxysmal atrial fibrillation induced by a focal discharge mechanism in the left superior pulmonary vein: possible roles of the ligament of Marshall. *J Cardiovasc Electrophysiol* 1999;10:636-648.

16. Katritsis D, Ioannidis JP, Anagnostopoulos CE, Sarris GE, Giazitzoglou E, Korovesis S, Camm AJ. Identification and catheter ablation of extracardiac and intracardiac components of ligament of Marshall tissue for treatment of paroxysmal atrial fibrillation. *J Cardiovasc Electrophysiol* 2001;12:750-758.

17. Polymeropoulos KP, Rodriguez LM, Timmermans C, Wellens HJ. Images in cardiovascular medicine. Radiofrequency ablation of a focal atrial tachycardia originating from the Marshall ligament as a trigger for atrial fibrillation. *Circulation* 2002;105:2112-2113.

18. Haemers P, Hamdi H, Guedj K, Suffee N, Farahmand P, Popovic N, Claus P, LePrince P, Nicoletti A, Jalife J, Wolke C, Lendeckel U, Jais P, Willems R, Hatem SN. Atrial fibrillation is associated with the fibrotic remodelling of adipose tissue in the

subepicardium of human and sheep atria. *Eur Heart J* 2017;38:53-61

19. Abouarab AA, Leonard JR, Ohmes LB, Lau C, Rong LQ, Ivascu NS, Pryor KO, Munjal M, Crea F, Massetti M, Sanna T, Girardi LN, Gaudino M. Posterior Left pericardiotomy for the prevention of postoperative Atrial fibrillation after Cardiac Surgery (PALACS): study protocol for a randomized controlled trial. *Trials* 2017;18:593.

20. Chan AW, Tetzlaff JM, Gotzsche PC, Altman DG, Mann H, Berlin JA, Dickersin K, Hrobjartsson A, Schulz KF, Parulekar WR, Krleza-Jeric K, Laupacis A, Moher D. SPIRIT 2013 explanation and elaboration: guidance for protocols of clinical trials. *BMJ* 2013;346:e7586.

21. Biancari F, Mahar MA. Meta-analysis of randomized trials on the efficacy of posterior pericardiotomy in preventing atrial fibrillation after coronary artery bypass surgery. J Thorac Cardiovasc Surg 2010;139:1158-1161.

pCAD-POAF Protocol changes from version 1.0 to version 2.0

Protocol amendment 1

Re: pCAD-POAF protocol version 2.0 (30th April 2021)

Previous version 1.0

4.2 Exclusion criteria

1) age > 75;

2) emergent CABG;

3) history of cardiac surgery;

4) EF < 40%;

5) Moderate of severer mitral regurgitation;

6) simultaneously undergoing any other cardiac surgery;

7) critical condition requiring hemodynamic support before CABG, such as need for extracorporeal membrane oxygenation or intra-aortic balloon pump;

8) history of AF last 6 months;

9) receiving antiarrhythmic therapies except of taking beta-blockers last 2 weeks before surgery.

10) Untreated hypothyroidism.

11) Serum creatinine > 150 umol/L.

Revised version 2.0

4.2 Exclusion criteria

1) age < 18;

2) emergent CABG;

3) history of cardiac surgery;

4) simultaneously undergoing any other cardiac surgery (such as Morrow procedure, valvular surgery, ventricular reconstruction due to ventricular aneurysm, or repair of the congenital heart diseases);

5) critical condition requiring hemodynamic support before CABG, such as need for extracorporeal membrane oxygenation or intra-aortic balloon pump;

6) history of AF;

7) receiving antiarrhythmic therapies except of taking beta-blockers last 2 weeks before surgery.

Partial CArdiac Denervation to Prevent PostOperative Atrial Fibrillation After Coronary Artery Bypass Grafting

（Study Number NCRC2020003)

Statistical Analysis Plan

(Version: 1.0)

Date 13 August 2022

Sign Off	Signature	Date
Professor<br>Wei Feng,<br>Chief Investigator	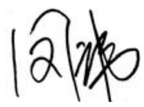	13 August 2022
Professor<br>Yang Wang,<br>Statistician	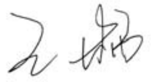	13 August 2022

Table of Contents

Title page 56

Abbreviation 59

1.Introduction 60

1.1 Background 60

1.2 Research hypothesis 62

1.3 Rationale of conducting this trial 62

1.4 Benefit /risk and ethical assessment 63

2. Study objective 63

2.1 Primary objective 63

2.2 Secondary objectives 64

2.2.1 Safety assessments 64

2.2.2 Economic assessments 64

3. Study design 64

3.1 Type of this study 64

3.2 Eligibility criteria 65

3.2.1 Inclusion criteria 65

3.2.2 Exclusion criteria 65

3.3 Overall study design and participants' timeline 65

3.4 Partial cardiac denervation procedure 67

3.5 Sample size justification 67

3.6 Randomization and blinding 68

3.6.1 Randomization after subjects enrolled ... 68

3.6.2 Blinding ... 68

4. Descriptive analyses ... 69

4.1 Study flow chart of the study ... 69

4.2 Baseline comparability of randomized groups ... 69

5. Patient groups for analysis of outcomes ... 70

5.1 Effectiveness ... 70

5.2 Safety ... 70

6. Statistical test for primary endpoint ... 70

6.1 Statistical analysis method ... 70

6.2 Missing data ... 71

7. Subgroup analysis for the primary outcome ... 71

8. Statistical test for secondary endpoints ... 72

8.1 Statistical analysis method ... 72

8.2 Missing data ... 72

9. Statistical analysis software ... 72

10. Interim analysis ... 73

Abbreviations

AF, atrial fibrillation

ANMTs, autonomic neuromodulation therapies

CABG, coronary artery bypass grafting

CANS, cardiac autonomic nerve system

ECG, electrocardiogram

GP, ganglionated plexi

LOM, ligament of Marshall

MACCE, major adverse cardiovascular and cerebrovascular events

POAF, postoperative atrial fibrillation

1.Introduction

1.1 Background

Postoperative atrial fibrillation (POAF) is one of the major complications after cardiac surgery, which occurs mostly within one week after the operation, with an incidence of 10–50%. POAF has been proven to increase the risk of hemodynamic deterioration, heart failure and stroke, resulting in increased hospital-stay as well as the medical expenses. While the incidence of POAF after coronary artery bypass grafting (CABG) ranges from 5% to 40%, it could result in severe postoperative circulatory fluctuations and expose patients to high risk of systemic embolism, such as stroke. Therefore, preventing POAF after CABG is essential.

The mechanism of POAF still remains unclear. Existing opinions are as follows: 1) disorder of cardiac autonomic nerve system (CANS), 2) inflammation, 3) oxidative stress, 4) abnormal activation of Ca^{+} channels, and 5) other potential mechanisms. Among these possible mechanisms, the activation of CANS plays an important role in the occurrence of POAF. In fact, beta-blocker, which mainly inhibits cardiac sympathetic excitability, is now the only drug listed as Class I recommendation by the present guideline to prevent POAF. However, previous study showed that even after administration of beta-blockers with a rate up to 80%, the incidence of POAF after CABG is as high as 21.1%.

Zafeiropoulos et al. meticulously summarized the potential role of autonomic neuromodulation therapies (ANMTs) in preventing POAF after cardiac surgery.

Studies also tried to reduce the incidence of POAF through surgical intervention of CANS during cardiac surgery, including resection of fat pads, ganglionated plexi (GP) ablation and botulinum toxin injection into epicardial fat pads. However, these studies differed in several aspects, including population size, eligibility criteria, randomization approach, surgical procedure and non-optimal electrocardiogram (ECG) monitoring strategies. As a result, they failed to reveal the potential benefit of ANMTs. More recently, Wang et al. reported a promising result of POAF reduction by Calcium Chloride injection into four major GPs during off-pump CABG (15% vs 36%, p=0.001), and the NeurOtoxin for the PreVention of Post-Operative Atrial Fibrillation trial also showed similar outcomes in isolated CABG patients, as well as in patients with advanced age receiving lower dose of botulinum toxin type A after cardiac surgery.

Partial cardiac denervation by resecting epicardial adipose tissue is also one of the ANMTs. Several previous studies evaluated the efficacy of ventral cardiac denervation through resecting fat pads surrounding the great vessels of heart on the prevention of POAF. Unfortunately, however, the results were inconsistent due to the heterogeneity of enrolled population, limited sample size and incomplete monitoring timeline.

1.2 Research hypothesis

This study is designed to evaluate the efficacy of partial cardiac denervation, which will be achieved by cutting off the ligament of Marshall (LOM) and resecting the fat pad along the Waterston groove, on the prevention of POAF.

1.3 Rationale of conducting this trial

As mentioned before, there is lack of specific recommendation of approach for preventing POAF after CABG except of beta-blockers and the related surgical means of ANMTs are still underestimated. Therefore, here in this study, we will focus on reevaluating the effect of partial cardiac denervation on preventing POAF after CABG in a larger population through more complete and longer duration of continuous ECG monitoring. To begin with, previous studies showed that the ligament of Marshall (LOM) is the critical site of CANS participating in the occurrence of atrial fibrillation (AF). Kim et al. noticed that the LOM contained sympathetic nerve fibers, and had insertions into the myocardium of the left atrium and coronary sinus, providing the essentials for the formation of arrythmia. Other reports clearly implicated the LOM and adjacent atrium as the origins of arrhythmias. In addition, Haemers et al. showed that right atrium was obviously infiltrated by adipose tissue in patients with AF, especially in those with persistent AF. Besides, cutting off the LOM and resecting the fat pad along the Waterston groove is routinely used in maze surgery to treat patients with AF. Lastly, this trial also aims to seek for a safe, simple and convenient way to prevent POAF after CABG. We believe that choosing one site at both right and left

atrium namely the LOM and the fat pad along the Waterston groove, exactly meets our purpose.

1.4 Benefit /risk and ethical assessment

The intervention group will receive additional partial cardiac denervation procedure, namely cutting off the LOM and resecting the fat pad along the Waterston groove. Potential risks are as follows: extending operation time, increasing intraoperative blood loss and postoperative arrhythmias. The related technique has been applied in surgical treatment of AF and proved to be safe and feasible. The surgery will remove only part of the CANS and will not affect the patient's normal sinus rhythm. All the participants can contact the study physician who will help them to get the right medical treatment, if any suspected arrhythmias-related adverse events emerge after surgery. The study will be approved by the Institutional Review Board of Fuwai Hospital.

2.Study objective

2.1 Primary objective

The primary outcome is POAF in 6 days, defined as a supraventricular arrhythmia lasting for >30 seconds. Patients will be monitored continuously beginning within one hour after the surgery to the 6th day postoperatively through the NS-SP-B-01 Attached Dynamic ECG Recording System. In addition, the overall lasting time of supraventricular arrhythmia will also be recorded for evaluating AF burden. Heart

rates will be continuously monitored, including lowest/highest/average heart beats, premature atrial/ventricular contractions etc. Two independent and blinded research doctors will interpret the ECG and determine the existence of POAF.

2.2 Secondary objectives

2.2.1 Safety assessments

1) The incidence of transferring to on-pump CABG intraoperatively;

2) The need for blood transfusion;

3) Re-operation for postoperative bleeding caused by partial cardiac denervation procedure;

4) The incidence of epicardial effusion within 30 days after discharge;

5) Arrhythmias other than AF within 30 days after discharge;

2.2.2 Economic assessments

6) Length of hospitalization;

7) All costs during hospitalization;

8) All costs after the surgery;

3. Study design

3.1 Type of this study

This is a single-centre, prospective, randomized, controlled study.

3.2 Eligibility criteria

We will consecutively recruit adult patients who are undergoing CABG (on-pump/off-pump) and agree to participate in this clinical trial.

3.2.1 Inclusion criteria

1)Adult patients scheduled for isolated CABG.

2)Patients who are willing to participate in this study and sign the informed consent.

3.2.2 Exclusion criteria

1) age < 18;

2) emergent CABG;

3) history of cardiac surgery;

4) simultaneously undergoing any other cardiac surgery (such as Morrow procedure, valvular surgery, ventricular reconstruction due to ventricular aneurysm, or repair of the congenital heart diseases);

5) critical condition requiring hemodynamic support before CABG, such as need for extracorporeal membrane oxygenation or intra-aortic balloon pump;

6) history of AF;

7) receiving antiarrhythmic therapies except of taking beta-blockers last 2 weeks before surgery.

3.3 Overall study design and participants' timeline

During the primary screening, patients will be evaluated for the eligibility into the study, and an individual informed consent will be signed at the willingness of each patient. Then, participants will be randomly allocated to either intervention or control group. After surgery, monitoring for the occurrence of AF will be lasted until the 6th day postoperatively among all of the patients. Meanwhile, complications such as pericardial effusion will also be assessed. On the day of discharge, participants will be investigated by 12-lead standard ECG and echocardiogram. During the 30-day follow-up, information such as the participants' overall health status, medication use, and whether they have experienced any kind of arrythmia or MACCEs, will be collected. The participants will also be investigated by ECG and echocardiogram again for further assessment (**Figure 1**).

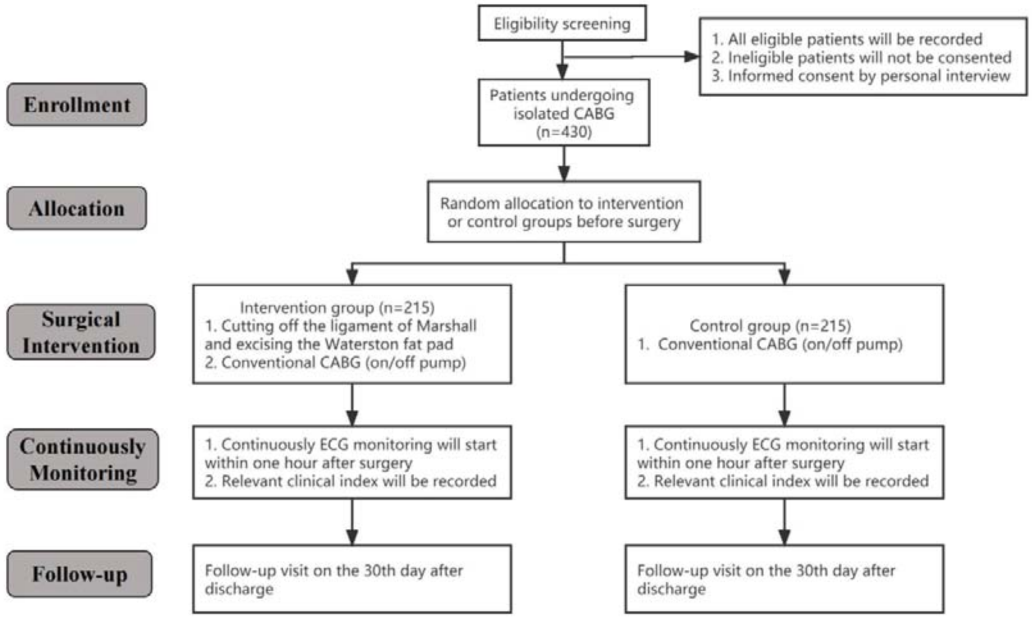

Figure 1. The study flow Chart.

3.4 Partial cardiac denervation procedure

In the intervention group, participant will undergo partial cardiac denervation through cutting off the LOM and resecting the fat pad along the Waterston groove. Specifically, during the on-pump CABG, the heart will be pulled to the right to expose the LOM between the left atrial appendage and the left pulmonary veins under extracorporeal circulation, and the LOM will be cut off by electrotome. Then, the fat pad along the Waterston groove will be exposed between the right pulmonary veins and right atrium and excised completely to the surface of myocardium, with the upper edge extending beyond the opening of the right upper pulmonary vein and the lower edge to the inferior vena cava. For off-pump CABG, we will cut off the LOM in the same way mentioned above. Then if the patient's heart is well tolerated and hemodynamics is stable, a fixator can be used to fix the heart when resecting the fat pad along the Waterston groove. Otherwise, cardiac denervation will be performed after CABG is completed. The estimated additional surgery time is 5 minutes. In order to achieve a satisfactory efficacy, a thorough cardiac denervation to myocardium surface will be emphasized. In addition, the existence of ganglia will be further proved through histologic analysis using the fat pad samples collected from 10 pairs of patients with or without POAF, respectively.

3.5 Sample size justification

Two parallel arms will be required. The occurrence of POAF after cardiac surgery was about 23%, according to the prior study. Based on a detailed reviewing of the existing

studies, cardiac denervation reduces the incidence of POAF by 50%. Therefore, with 80% power and 0.05 alpha, 408 participants are needed to detect 23% POAF rate in the control group and a reduction by 50% in the intervention group. Taking into account a 5% protocol violation rate and patient loss/dropout, a population size of 430 (215 in each group) shall be sufficient for this investigation. As one of the world's largest centers of cardiovascular diseases, Fuwai Hospital completes more than 10 thousand cardiac surgeries every year, of which an approximate of 5 thousand are isolated CABG. This trial shall be completed in due course.

3.6 Randomization and blinding

3.6.1 Randomization after subjects enrolled

A controlled, randomized process will be used to assign participants to treatment groups. A computer-generated minimized random allocation technique based on factors including age, sex, history of myocardial infarction, and left ventricular ejection fraction will be employed to ensure that the cases are distributed equally. The department research fellow will inform the surgeon of the patient's group assignment once they are under general anesthesia in the operating room.

3.6.2 Blinding

As different surgical operations are involved, complete double-blindness cannot be achieved. In order to reduce the researcher bias, surgeons are not allowed to know the result of patient's assignment until surgery, and the third-party supervisor responsible

for randomization shall inform the surgeons of the results only after general anesthesia and before the surgical procedure starts. Patients and data analysts will be blinded throughout the process. Unblinding will be done under the third-party supervision after all data analysis is completed.

4. Descriptive analyses

4.1 Study flow chart of the study

A CONSORT flow diagram will be presented showing the numbers of patients screened, randomized, received surgery (with details), and followed up after discharge.

4.2 Baseline comparability of randomized groups

Demographic and clinical characteristics of patients at baseline will be presented by randomised group. Number of subjects, mean, standard deviation, median, interquartile range (IQR), minimum, and maximum will be used for description of continuous variables. Continuous variables will be tested by Student's t test if normally distributed; otherwise, by the Mann-Whitney U test. Counts and percentages will be used to summarize the categorical variables. Chi-squared test or Fisher's exact test will be applied for categorical variables, as appropriate.

Intraoperative data such as number of grafts, % off pump, % saphenous vein/radial artery, duration of operation will also be presented.

5. Patient groups for analysis of outcomes

5.1 Effectiveness

The main analyses will be conducted on all randomized patients, applying the principle of intention to treat (ITT), as far as is practically possible, except where specified. The only reason for excluding patients will be the absence of outcome information. All patients randomized will be analysed in the groups assigned at randomization, irrespective of subsequent management and events. Thus any patients deemed ineligible following randomization will not be removed from the trial if this information became available after randomization and thus after disclosure of the allocation.

5.2 Safety

In the analysis of safety/harms patients will be analysed both by ITT and according to the operation received (i.e. per-protocol). Only patients who received the surgical procedure to which they were randomly allocated will be included in the per protocol analysis of safety data.

6. Statistical test for primary endpoint

6.1 Statistical analysis method

The primary endpoint is the POAF incidence in 6 days after surgery. For the primary

outcome, results will be compared as categorical variable and according to the approach mentioned in 4.2. As sensitivity analysis of the primary outcome, the time-to-event analysis will be performed with Kaplan-Meier survival curves and compared by the log-rank test. In addition, the AF episodes, AF burden, lowest/highest/average heart beats and premature atrial contractions will also be compared by approach mentioned in 4.2. Unless otherwise stated, statistical significance level is set to 0.05 (2-sided).

6.2 Missing data

Since the POAF occurrence will be obtained from monitoring devices in participant's hospitalization, missing data won't be expected.

7. Subgroup analysis for the primary outcome

Subgroup analysis will be conducted based on the risk factors of POAF reported by previous studies as follows: sex, age (≥ 65 years vs. < 65 years), LVEF (> 55% vs. ≤ 55%), body mass index (≥ 25 kg/m^2 vs. < 25 kg/m^2), left atrium size (≥ 40 mm vs. < 40 mm), and history of myocardial infarction, diabetes mellitus and hypertension.

To test for a differential effect of randomized treatment across subgroups, an interaction term (treatment group by subgroup) will be fitted in the Cox proportional hazards model. If proportional hazards cannot be assumed a multiple logistic regression model will be fitted.

Subgroup analyses will be conducted only with respect to the primary outcome.

Treatment effect in each subgroup shall be presented along with the P value for the interaction term in the model.

All patients randomized shall be included in the analysis, irrespective of whether surgery was undertaken.

8. Statistical test for secondary endpoints

8.1 Statistical analysis method

Secondary outcomes including safety and economics will also be analyzed according to approach mentioned in 4.2. Unless otherwise stated, statistical significance level is set to 0.05 (2-sided).

8.2 Missing data

Since the secondary endpoints are events occurring either in participant's hospitalization or a relatively short follow-up visit (30 days after discharge), missing data shall not be expected. Any missing data won't be imputed.

9. Statistical analysis software

Statistical analyses will be performed by using R 4.0.2 (R Core Team, Vienna, Austria) and Stata 15.0 (StataCorp，College Station，TX，USA).

10. Interim analysis

No interim analysis for this study.

Partial CArdiac Denervation to Prevent PostOperative Atrial Fibrillation After Coronary Artery Bypass Grafting

（Study Number NCRC2020003)

Statistical Analysis Plan

(Version: 3.0)

Date 08 May 2024

Sign Off	Signature	Date
Professor<br>Wei Feng,<br>Chief Investigator		08 May 2024
Professor<br>Yang Wang,<br>Statistician		08 May 2024

Table of Contents

Title page 74

Abbreviation 77

1.Introduction 78

1.1 Background 78

1.2 Research hypothesis 80

1.3 Rationale of conducting this trial 80

1.4 Benefit /risk and ethical assessment 81

2. Study objective 81

2.1 Primary objective 81

2.2 Secondary objectives 82

2.2.1 Safety assessments 82

2.2.2 Economic assessments 82

3. Study design 82

3.1 Type of this study 82

3.2 Eligibility criteria 83

3.2.1 Inclusion criteria 83

3.2.2 Exclusion criteria 83

3.3 Overall study design and participants' timeline 84

3.4 Partial cardiac denervation procedure 85

3.5 Sample size justification 85

3.6 Randomization and blinding 86

3.6.1 Randomization after subjects enrolled 86
3.6.2 Blinding 86
4. Descriptive analyses 87
4.1 Study flow chart of the study 87
4.2 Baseline comparability of randomized groups 87
5. Patient groups for analysis of outcomes 88
5.1 Effectiveness 88
5.2 Safety 88
6. Statistical test for primary endpoint 88
6.1 Statistical analysis method 88
6.2 Missing data 89
7. Subgroup analysis for the primary outcome 89
8. Statistical test for secondary endpoints 90
8.1 Statistical analysis method 90
8.2 Missing data 91
9. Statistical analysis software 91
10. Interim analysis 91

Abbreviations

AF, atrial fibrillation

ANMTs, autonomic neuromodulation therapies

CABG, coronary artery bypass grafting

CANS, cardiac autonomic nerve system

ECG, electrocardiogram

GP, ganglionated plexi

LOM, ligament of Marshall

MACCE, major adverse cardiovascular and cerebrovascular events

POAF, postoperative atrial fibrillation

1.Introduction

1.1 Background

Postoperative atrial fibrillation (POAF) is one of the major complications after cardiac surgery, which occurs mostly within one week after the operation, with an incidence of 10–50%. POAF has been proven to increase the risk of hemodynamic deterioration, heart failure and stroke, resulting in increased hospital-stay as well as the medical expenses. While the incidence of POAF after coronary artery bypass grafting (CABG) ranges from 5% to 40%, it could result in severe postoperative circulatory fluctuations and expose patients to high risk of systemic embolism, such as stroke. Therefore, preventing POAF after CABG is essential.

The mechanism of POAF still remains unclear. Existing opinions are as follows: 1) disorder of cardiac autonomic nerve system (CANS), 2) inflammation, 3) oxidative stress, 4) abnormal activation of Ca^{+} channels, and 5) other potential mechanisms. Among these possible mechanisms, the activation of CANS plays an important role in the occurrence of POAF. In fact, beta-blocker, which mainly inhibits cardiac sympathetic excitability, is now the only drug listed as Class I recommendation by the present guideline to prevent POAF. However, previous study showed that even after administration of beta-blockers with a rate up to 80%, the incidence of POAF after CABG is as high as 21.1%.

Zafeiropoulos et al. meticulously summarized the potential role of autonomic neuromodulation therapies (ANMTs) in preventing POAF after cardiac surgery.

Studies also tried to reduce the incidence of POAF through surgical intervention of CANS during cardiac surgery, including resection of fat pads, ganglionated plexi (GP) ablation and botulinum toxin injection into epicardial fat pads. However, these studies differed in several aspects, including population size, eligibility criteria, randomization approach, surgical procedure and non-optimal electrocardiogram (ECG) monitoring strategies. As a result, they failed to reveal the potential benefit of ANMTs. More recently, Wang et al. reported a promising result of POAF reduction by Calcium Chloride injection into four major GPs during off-pump CABG (15% vs 36%, p=0.001), and the NeurOtoxin for the PreVention of Post-Operative Atrial Fibrillation trial also showed similar outcomes in isolated CABG patients, as well as in patients with advanced age receiving lower dose of botulinum toxin type A after cardiac surgery.

Partial cardiac denervation by resecting epicardial adipose tissue is also one of the ANMTs. Several previous studies evaluated the efficacy of ventral cardiac denervation through resecting fat pads surrounding the great vessels of heart on the prevention of POAF. Unfortunately, however, the results were inconsistent due to the heterogeneity of enrolled population, limited sample size and incomplete monitoring timeline.

1.2 Research hypothesis

This study is designed to evaluate the efficacy of partial cardiac denervation, which will be achieved by cutting off the ligament of Marshall (LOM) and resecting the fat pad along the Waterston groove, on the prevention of POAF.

1.3 Rationale of conducting this trial

As mentioned before, there is lack of specific recommendation of approach for preventing POAF after CABG except of beta-blockers and the related surgical means of ANMTs are still underestimated. Therefore, here in this study, we will focus on reevaluating the effect of partial cardiac denervation on preventing POAF after CABG in a larger population through more complete and longer duration of continuous ECG monitoring. To begin with, previous studies showed that the ligament of Marshall (LOM) is the critical site of CANS participating in the occurrence of atrial fibrillation (AF). Kim et al. noticed that the LOM contained sympathetic nerve fibers, and had insertions into the myocardium of the left atrium and coronary sinus, providing the essentials for the formation of arrythmia. Other reports clearly implicated the LOM and adjacent atrium as the origins of arrhythmias. In addition, Haemers et al. showed that right atrium was obviously infiltrated by adipose tissue in patients with AF, especially in those with persistent AF. Besides, cutting off the LOM and resecting the fat pad along the Waterston groove is routinely used in maze surgery to treat patients with AF. Lastly, this trial also aims to seek for a safe, simple and convenient way to prevent POAF after CABG. We believe that choosing one site at both right and left

atrium namely the LOM and the fat pad along the Waterston groove, exactly meets our purpose.

1.4 Benefit /risk and ethical assessment

The intervention group will receive additional partial cardiac denervation procedure, namely cutting off the LOM and resecting the fat pad along the Waterston groove. Potential risks are as follows: extending operation time, increasing intraoperative blood loss and postoperative arrhythmias. The related technique has been applied in surgical treatment of AF and proved to be safe and feasible. The surgery will remove only part of the CANS and will not affect the patient's normal sinus rhythm. All the participants can contact the the study physician who will help them to get the right medical treatment, if any suspected arrhythmias-related adverse events emerge after surgery. The study will be approved by the Institutional Review Board of Fuwai Hospital.

2. Study objective

2.1 Primary objective

The primary outcome is POAF in 6 days, defined as a supraventricular arrhythmia lasting for >30 seconds. Patients will be monitored continuously beginning within one hour after the surgery to the 6th day postoperatively through the NS-SP-B-01 Attached Dynamic ECG Recording System. In addition, the overall lasting time of supraventricular arrhythmia will also be recorded for evaluating AF burden. Heart

rates will be continuously monitored, including lowest/highest/average heart beats, premature atrial/ventricular contractions etc. Two independent and blinded research doctors will interpret the ECG and determine the existence of POAF.

2.2 Secondary objectives

2.2.1 Safety assessments

1) The incidence of transferring to on-pump CABG intraoperatively;

2) The need for blood transfusion;

3) Re-operation for postoperative bleeding caused by partial cardiac denervation procedure;

4) The incidence of epicardial effusion within 30 days after discharge;

5) Arrhythmias other than AF within 30 days after discharge;

2.2.2 Economic assessments

6) Length of hospitalization;

7) All costs during hospitalization;

8) All costs after the surgery;

3. Study design

3.1 Type of this study

This is a single-centre, prospective, randomized, controlled study.

3.2 Eligibility criteria

We will consecutively recruit adult patients who are undergoing CABG (on-pump/off-pump) and agree to participate in this clinical trial.

3.2.1 Inclusion criteria

1)Adult patients scheduled for isolated CABG.

2)Patients who are willing to participate in this study and sign the informed consent.

3.2.2 Exclusion criteria

1) age < 18;

2) emergent CABG;

3) history of cardiac surgery;

4) simultaneously undergoing any other cardiac surgery (such as Morrow procedure, valvular surgery, ventricular reconstruction due to ventricular aneurysm, or repair of the congenital heart diseases);

5) critical condition requiring hemodynamic support before CABG, such as need for extracorporeal membrane oxygenation or intra-aortic balloon pump;

6) history of AF;

7) receiving antiarrhythmic therapies except of taking beta-blockers last 2 weeks before surgery.

3.3 Overall study design and participants' timeline

During the primary screening, patients will be evaluated for the eligibility into the study, and an individual informed consent will be signed at the willingness of each patient. Then, participants will be randomly allocated to either intervention or control group. After surgery, monitoring for the occurrence of AF will be lasted until the 6th day postoperatively among all of the patients. Meanwhile, complications such as pericardial effusion will also be assessed. On the day of discharge, participants will be investigated by 12-lead standard ECG and echocardiogram. During the 30-day follow-up, information such as the participants' overall health status, medication use, and whether they have experienced any kind of arrythmia or MACCEs, will be collected. The participants will also be investigated by ECG and echocardiogram again for further assessment (**Figure 1**).

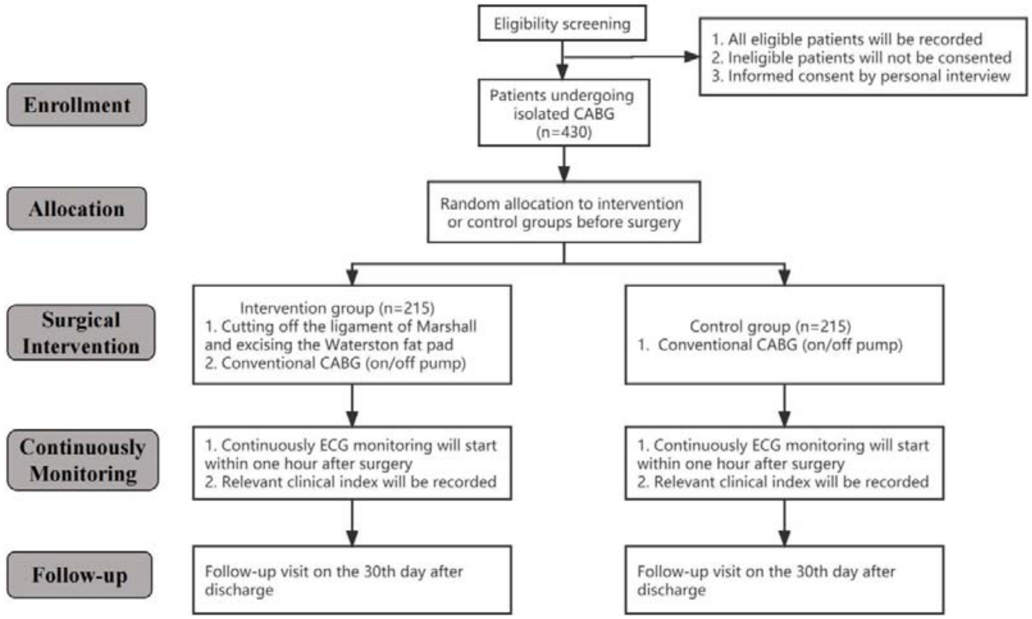

Figure 1. The study flow Chart.

3.4 Partial cardiac denervation procedure

In the intervention group, participant will undergo partial cardiac denervation through cutting off the LOM and resecting the fat pad along the Waterston groove. Specifically, during the on-pump CABG, the heart will be pulled to the right to expose the LOM between the left atrial appendage and the left pulmonary veins under extracorporeal circulation, and the LOM will be cut off by electrotome. Then, the fat pad along the Waterston groove will be exposed between the right pulmonary veins and right atrium and excised completely to the surface of myocardium, with the upper edge extending beyond the opening of the right upper pulmonary vein and the lower edge to the inferior vena cava. For off-pump CABG, we will cut off the LOM in the same way mentioned above. Then if the patient's heart is well tolerated and hemodynamics is stable, a fixator can be used to fix the heart when resecting the fat pad along the Waterston groove. Otherwise, cardiac denervation will be performed after CABG is completed. The estimated additional surgery time is 5 minutes. In order to achieve a satisfactory efficacy, a thorough cardiac denervation to myocardium surface will be emphasized. In addition, the existence of ganglia will be further proved through histologic analysis using the fat pad samples collected from 10 pairs of patients with or without POAF, respectively.

3.5 Sample size justification

Two parallel arms will be required. The occurrence of POAF after cardiac surgery was about 23%, according to the prior study. Based on a detailed reviewing of the existing

studies, cardiac denervation reduces the incidence of POAF by 50%. Therefore, with 80% power and 0.05 alpha, 408 participants are needed to detect 23% POAF rate in the control group and a reduction by 50% in the intervention group. Taking into account a 5% protocol violation rate and patient loss/dropout, a population size of 430 (215 in each group) shall be sufficient for this investigation. As one of the world's largest centers of cardiovascular diseases, Fuwai Hospital completes more than 10 thousand cardiac surgeries every year, of which an approximate of 5 thousand are isolated CABG. This trial shall be completed in due course.

3.6 Randomization and blinding

3.6.1 Randomization after subjects enrolled

A controlled, randomized process will be used to assign participants to treatment groups. A computer-generated minimized random allocation technique based on factors including age, sex, history of myocardial infarction, and left ventricular ejection fraction will be employed to ensure that the cases are distributed equally. The department research fellow will inform the surgeon of the patient's group assignment once they are under general anesthesia in the operating room.

3.6.2 Blinding

As different surgical operations are involved, complete double-blindness cannot be achieved. In order to reduce the researcher bias, surgeons are not allowed to know the result of patient's assignment until surgery, and the third-party supervisor responsible

for randomization shall inform the surgeons of the results only after general anesthesia and before the surgical procedure starts. Patients and data analysts will be blinded throughout the process. Unblinding will be done under the third-party supervision after all data analysis is completed.

4. Descriptive analyses

4.1 Study flow chart of the study

A CONSORT flow diagram will be presented showing the numbers of patients screened, randomized, received surgery (with details), and followed up after discharge.

4.2 Baseline comparability of randomized groups

Demographic and clinical characteristics of patients at baseline will be presented by randomised group. Number of subjects, mean, standard deviation, median, interquartile range (IQR), minimum, and maximum will be used for description of continuous variables. Continuous variables will be tested by Student's t test if normally distributed; otherwise, by the Mann-Whitney U test. Counts and percentages will be used to summarize the categorical variables. Chi-squared test or Fisher's exact test will be applied for categorical variables, as appropriate.

Intraoperative data such as number of grafts, % off pump, % saphenous vein/radial artery, duration of operation will also be presented.

5.Patient groups for analysis of outcomes

5.1 Effectiveness

The main analyses will be conducted on all randomized patients, applying the principle of intention to treat (ITT), as far as is practically possible, except where specified. The only reason for excluding patients will be the absence of outcome information. All patients randomized will be analysed in the groups assigned at randomization, irrespective of subsequent management and events. Thus any patients deemed ineligible following randomization will not be removed from the trial if this information became available after randomization and thus after disclosure of the allocation.

5.2 Safety

In the analysis of safety/harms patients will be analysed both by ITT and according to the operation received (i.e. per-protocol). Only patients who received the surgical procedure to which they were randomly allocated will be included in the per protocol analysis of safety data.

6. Statistical test for primary endpoint

6.1 Statistical analysis method

The primary endpoint is the POAF incidence in 6 days after surgery. For the primary

outcome, results will be compared as categorical variable and according to the approach mentioned in 4.2. As sensitivity analysis of the primary outcome, the time-to-event analysis will be performed with Kaplan-Meier survival curves and compared by the log-rank test. In addition, the AF episodes, AF burden, lowest/highest/average heart beats and premature atrial contractions will also be compared by approach mentioned in 4.2. Unless otherwise stated, statistical significance level is set to 0.05 (2-sided).

6.2 Missing data

Since the POAF occurrence will be obtained from monitoring devices in participant's hospitalization, missing data won't be expected.

7. Subgroup analysis for the primary outcome

Subgroup analysis will be conducted based on the risk factors of POAF reported by previous studies as follows: sex, age (≥ 65 years vs. < 65 years), LVEF (> 55% vs. ≤ 55%), body mass index (≥ 25 kg/m^2 vs. < 25 kg/m^2), left atrium size (≥ 40 mm vs. < 40 mm), and history of myocardial infarction, diabetes mellitus and hypertension.

To test for a differential effect of randomized treatment across subgroups, an interaction term (treatment group by subgroup) will be fitted in the Cox proportional hazards model. If proportional hazards cannot be assumed a multiple logistic regression model will be fitted.

Subgroup analyses will be conducted only with respect to the primary outcome.

Treatment effect in each subgroup shall be presented along with the P value for the interaction term in the model.

All patients randomized shall be included in the analysis, irrespective of whether surgery was undertaken.

8. Statistical test for secondary endpoints

8.1 Statistical analysis method

Secondary outcomes including safety and economics will also be analyzed according to approach mentioned in 4.2. Unless otherwise stated, statistical significance level is set to 0.05 (2-sided).

For clinical concerns, the incidence of epicardial effusion within 30 days after discharge and arrhythmias other than AF within 30 days after discharge will be analysed as delayed epicardial effusion and critical arrhythmias, respectively. Delayed epicardial effusion was defined as new-onset epicardial effusion (moderate or more) within 30 days after discharge. Critical arrhythmias other than AF was defined as arrhythmias requiring immediate clinical intervention, such as complete atrioventricular block.

Since POAF happens after surgery and has a effect on postoperative clinical events, we think the analysis of all cost during hospitalization may not be appropriate. Only postoperative cost for each patient will be included in the final analysis. Similarly, only postoperative length of hospitalization will be included in the final analysis.

Although the follow-up visit of 30 days is relative short, we intend to compare the MACCE outcome between the 2 groups in the final manuscript.

8.2 Missing data

Since the secondary endpoints are events occurring either in participant's hospitalization or a relatively short follow-up visit (30 days after discharge), missing data shall not be expected. Any missing data won't be imputed.

9. Statistical analysis software

Statistical analyses will be performed by using R 4.0.2 (R Core Team, Vienna, Austria) and Stata 15.0 (StataCorp，College Station，TX，USA).

10.Interim analysis

No interim analysis for this study.

Summary of changes of the Statistical Analysis Plan from version 1.0 to version 3.0

For clinical concerns, the incidence of epicardial effusion within 30 days after discharge and arrhythmias other than AF within 30 days after discharge will be analysed as delayed epicardial effusion and critical arrhythmias, respectively. Delayed epicardial effusion was defined as new-onset epicardial effusion (moderate or more) within 30 days after discharge. Critical arrhythmias other than AF was defined as arrhythmias requiring immediate clinical intervention, such as complete atrioventricular block.

Since POAF happens after surgery and has a effect on postoperative clinical events, we think the analysis of all cost during hospitalization may not be appropriate. Only postoperative cost for each patient will be included in the final analysis. Similarly, only postoperative length of hospitalization will be included in the final analysis.

Although the follow-up visit of 30 days is relative short, we intend to compare the MACCE outcome between the 2 groups in the final manuscript.
