# Supplementary material for: Partial Cardiac Denervation to Prevent Postoperative Atrial Fibrillation After Coronary Artery Bypass Grafting: The pCAD-POAF Randomized Clinical Trial
Source: JAMA Cardiol. 2024 Nov 17:e244639. Online ahead of print. doi: 10.1001/jamacardio.2024.4639 (PMC11571071; doi:10.1001/jamacardio.2024.4639)
Supplement: Supplement 3. — Data Sharing Statement [file jamacardiol-e244639-s003.pdf]

Data Sharing Statement

Yang. Partial Cardiac Denervation to Prevent Postoperative Atrial Fibrillation After Coronary Artery Bypass Grafting. *JAMA Cardiol*. Published November 17, 2024. doi:10.1001/jamacardio.2024.4639

Data
Additional Information: Trial Registration: ClinicalTrials.gov Identifier: NCT05009914
Data available: No

Additional Information
Explanation for why data not available: All reasonable data request shall be submitted to the corresponding author (Wei Feng) for consideration.
